# Dual-Coordinate Relative Free Energy Simulations Using Machine-Learned Interatomic Potentials

**DOI:** 10.1021/acs.jctc.6c00824

**Published:** 2026-07-22

**Authors:** Anna Katharina Picha, Stefan Boresch

**Affiliations:** † 27258 University of Vienna, Faculty of Chemistry, Institute of Computational Biological Chemistry, Vienna 1090, Austria; ‡ University of Vienna, Vienna Doctoral School of Chemistry (DosChem), Vienna 1090, Austria

## Abstract

The integration of machine-learned interatomic potentials (MLIPs) into free energy simulations (FES) offers the promise of near-quantum mechanical accuracy at a reduced computational cost. However, employing MLIPs for alchemical relative free energy calculations remains challenging since MLIPs are typically not trained to handle the unphysical intermediate states required for such transformations. In previous work, we demonstrated how atoms and molecules can be gradually decoupled in systems fully described by MLIPs by manipulating the neighbor list and introducing an artificial offset to interatomic distances. Here, we extend this approach to general alchemical transformations between two states and apply the methodology to computing relative solvation free energies (RSFEs) between arbitrary solute pairs in systems fully described by an unmodified MLIP. We employ a dual-coordinate approach where both solutes are explicitly present but do not interact with one another. By introducing λ-dependent distance offsets to the neighbor list, we perform a simultaneous transformation: smoothly decoupling the first solute from the solvent while coupling the second solute to the same environment. To enforce spatial overlap without modifying the internal intramolecular dynamics of either solute, we utilize a harmonic ″anchor″ restraint to a central atom on each molecule. We demonstrate the robustness of this method using the MACE-OFF23­(S) potential across a diverse set of small molecule pairs, including transformations between species with no chemical similarity (e.g., toluene to tetrahydrofuran). The method’s accuracy is validated through thermodynamic cycle closure by combining direct RSFE calculations with absolute solvation free energy (ASFE) calculations. The methodology achieves excellent internal consistency, with cycle closure errors less than or equal to ±0.11 kcal/mol, well within the estimated statistical uncertainty. By requiring only a single energy evaluation per simulation step and avoiding architecture-specific modifications and retraining, this anchor-based dual-coordinate approach provides a highly adaptable and efficient route for relative alchemical FES with modern MLIPs.

## Graphical abstract

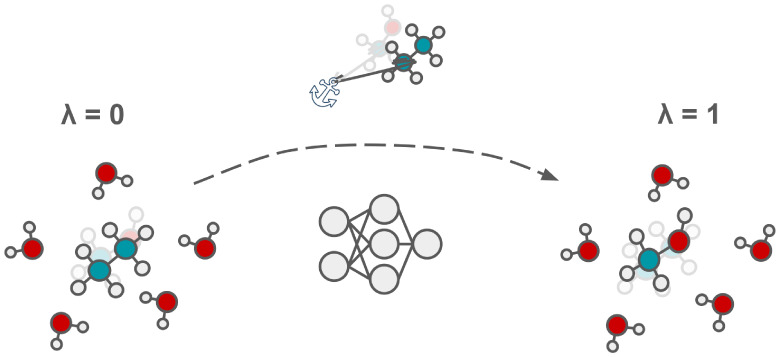

## Introduction

The free energy difference, Δ*G*, between two chemical systems is the central thermodynamic quantity that determines relative stability of two states, as well as the directionality of chemical processes and reactions. Consequently, the calculation of free energy differences by using molecular dynamics (MD)-based free energy simulations (FES) has become an indispensable tool in computational chemistry, biology, materials science, and drug development; see, e.g., refs 1–6 . Prominent applications include the prediction of solvation free energy differences^7–9^ partition coefficients^9,10^ and, most notably, binding free energies, which are now routinely employed in structure-based drug design and lead optimization workflows.^11–13^ In many applications, it is sufficient to know relative free energy differences ΔΔ*G*, e.g., the relative difference in binding free energy between two ligands or in solvation free energy between two solutes. Exploiting thermodynamic cycles,^14^ the ΔΔ*G* of interest can be computed either along chemical or alchemical paths. Whether one route is computationally advantageous—or even feasible—depends on the degree of similarity (or dissimilarity) between the two end states, such as the two ligands or solutes. Therefore, FES workflows need to have efficient methods to compute free energy differences along both chemical and alchemical paths.

The vast majority of FES employs classical force fields. Their approximate nature ultimately limits the attainable accuracy of the resulting (Δ)­Δ*G* values. Although quantum mechanical (QM) or hybrid QM/MM Hamiltonians could, in principle, provide a more faithful description of the underlying physical behavior, their substantially higher computational cost renders routine free energy simulations impractical for many systems of practical interest. In recent years, machine-learned interatomic potentials (MLIPs) have emerged as a promising alternative to classical force fields. Examples include ANI^15,16^ SchNet,^17^ PaiNN,^18^ PhysNet,^19^ DimeNet/DimeNet^+2^ ^20,21^ SpookyNet,^22^ NequIP,^23^ MACE,^24^ GRACE,^25^ AIMNet2,^26^ among others. Similarly, a growing number of data sets have become available for training these models.^16,27–29^ Trained on large data sets of high-level quantum mechanical (QM) energies and forces, MLIPs can reproduce near-QM accuracy at a fraction of the computational cost.^30,31^ MLIPs—and hybrid MLIP/MM approaches—enable MD simulations of condensed-phase systems such as solvated small molecules or protein–ligand complexes on nanosecond time scales^16,24,32,33^ which suggests the combination with FES. However, the calculation of free energy differences in combination with MLIPs is in the early stages of development, and several well-established “tricks of the trade” will need to be adapted when using MLIPs.

Alchemical free energy methods rely on nonphysical intermediate states, whereas QM Hamiltonians—and, by extension, MLIPs trained on QM data—are well-defined only for physically admissible states. For example, configurations involving tri- or pentavalent carbon atoms are unphysical and cannot be represented by standard QM methods or MLIPs without additional corresponding training. When QM/MM Hamiltonians are used in FES, these limitations are typically circumvented through so-called indirect approaches,^34^ which have also been adopted in the context of MLIP/MM simulations.^35–37^ Very recently, Xie et al. proposed a related approach where solute–solvent interactions are first switched to a force field description and the alchemical transformation is done at the force field level of theory.^38^ While effective, such strategies add conceptual and computational complexity. Therefore, direct alchemical transformations using MLIPs would be highly desirable.

Recently, several approaches have been reported that enable coupling and decoupling processes in systems fully described by MLIPs, thereby allowing for the computation of absolute free energy differences. Some of these strategies rely on targeted modifications of the neural network architecture, e.g., its underlying message-passing scheme. For example, Harry Moore et al. introduced architecture-specific adaptations to the MACE potential to permit the decoupling of solute molecules from the surrounding solvent,^39^ with related concepts also reported by Nam et al.^40^ Plé et al. reported absolute solvation free energy (ASFE) calculations by scaling pairwise cutoff functions.^41^ While these approaches are, in principle, transferable to other MLIP frameworks, they require nontrivial and architecture-dependent modifications. Moreover, to date, they have primarily been demonstrated for decoupling transformations only and, thus, for calculations of absolute free energies, i.e., FES along chemical paths. Recently, we introduced an approach based on manipulating the neighbor list and introducing artificial distance offsets in MLIP-driven MD simulations to gradually decouple a solute from its surrounding solvent.^42^ By selectively controlling which atomic interactions are considered by the neural network, this method enables smooth, nonphysical transformations without modifying the MLIP itself. This method allowed for an efficient computation of ASFEs and is, in theory, agnostic to the underlying MLIP architecture. We refer to this approach as *4D-decoupling*.

In contrast, a general strategy enabling direct alchemical transformations remains an open challenge, and only a limited number of studies have explored relative free energy calculations with MLIPs. Tkaczyk et al.^43^ employed interpolation-based schemes in which multiple Hamiltonians must be evaluated for each configuration. As a consequence, this approach entails a substantially increased computational cost, since the energy must be evaluated twice per simulation step. Moreover, the interpolation framework is primarily applicable to solute pairs exhibiting a high degree of structural similarity (e.g., tautomers). Crha et al.^44^ demonstrated the computation of relative free energy differences using a custom-trained MLIP. Their approach was only illustrated for a single solute pair, and the simulated system was described only partially by the MLIP. An alternative strategy, the alchemical transfer method (ATM)^45,46^ can in principle be applied to MLIPs without modifying the potential itself, but ATM requires substantially larger system sizes and is not suitable for the calculation of solvation free energies. To facilitate comparison, we summarize all described methods in Table S1 in the Supporting Information.

In this work, we build upon our previously introduced neighbor list manipulation strategy and present a general framework for performing direct alchemical transformations between—in principle—arbitrary pairs of small solutes in aqueous solution using systems described entirely by an unmodified MLIP. By extending the concept of interaction decoupling to solute–solute transformations through additional manipulations of the interatomic pairwise distance information passed to the MLIP, we enable relative alchemical free energy calculations without modifying the underlying neural network architecture and without requiring a predefined degree of chemical similarity between the transformed molecules. Consequently, relative solvation free energies (RSFEs) can be computed for arbitrary solute pairs. Importantly, the proposed approach does not require multiple energy evaluations per simulation step and therefore provides a computationally efficient route to the direct calculation of RSFEs. When combined with our earlier method for computing ASFEs, the framework can be validated via thermodynamic cycle closure. We assess the methodology for several solute pairs, report relative free energy differences and demonstrate cycle closure obtained with the MACE-OFF23­(S) MLIP.

The remainder of this manuscript is organized as follows: In the Theory section, we give an overview of the most important theoretical concepts used in this study. To understand the methodological challenges, we first revisit the technical requirements to carry out FES along chemical and alchemical paths, respectively. Second, we briefly describe the distance-dependent information flow in MLIPs. Third, we introduce the dual-coordinate paradigm used and describe how solutes are coupled and decoupled from the surrounding solvent. Fourth, we describe our methodology for applying positional restraints to the two solutes that enforces their spatial colocalization within the same region and interaction with mostly the same solvent molecules by restraining the position of a single atom in each solute. Fifth, we describe how we exploit cycle closure in our dual-coordinate approach. In the Methods section, we briefly introduce the set of solutes and solute pairs for which absolute and relative free energy differences in aqueous solution were computed and summarize the simulation setup and the protocols employed for the free energy calculations. In the Results section, we report ASFEs and RSFEs and assess thermodynamic cycle closure. Finally, in the Summary and Outlook section, we recapitulate our main findings, discuss methodological limitations and practical considerations of the proposed approach, and outline possible directions for future methodological refinements and extensions.

## Theory

### FES and Thermodynamic Cycles

Consider the thermodynamic cycle shown in Figure 1, concerned with the ASFEs $\Delta G_{\mathrm{solv}}^{S1}$ and $\Delta G_{\mathrm{solv}}^{S2}$ of two solutes, *S*1 and *S*2 (vertical arrows). Since these are the quantities which, in principle, can be measured, we refer to these paths as *physical* or *chemical* ones.

**Figure 1:**
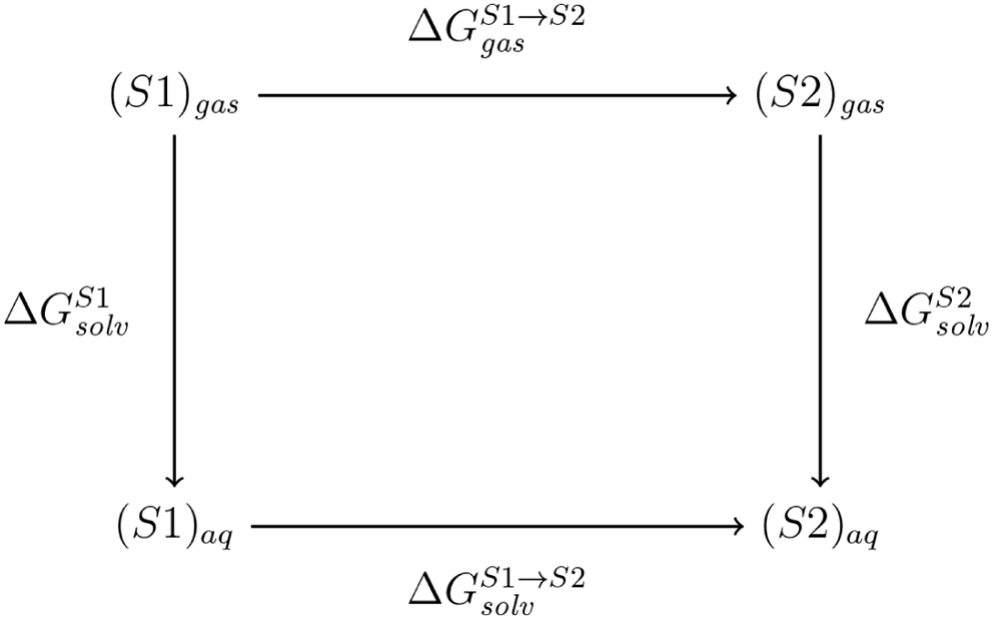
Schematic thermodynamic cycle to calculate RSFE differences: $\Delta G_{\mathrm{gas}}^{S1\to S2}$ denotes the free energy difference between the two solutes, *S*1 and *S*2, in the gas phase, whereas $\Delta G_{\mathrm{solv}}^{S1\to S2}$ represents the corresponding free energy difference in aqueous solution. $\Delta G_{\mathrm{solv}}^{S1}$ and $\Delta G_{\mathrm{solv}}^{S2}$ denote the ASFE for *S*1 and *S*2, respectively.

Often, one does not need to know the ASFEs of the individual compounds, but is only interested in the RSFE difference between *S*1 and *S*2. In other words, one wants to know the double free energy difference $\Delta \Delta G_{\mathrm{solv}}=\Delta G_{\mathrm{solv}}^{S2}-\Delta G_{\mathrm{solv}}^{S1}$ . Of course, one can compute each of the two ASFEs and take the difference. Alternatively, one can exploit the path independence of the free energy,^14^ i.e.,

1 $$\Delta G_{\mathrm{gas}}^{S1\to S2}+\Delta G_{\mathrm{solv}}^{S2}-\Delta G_{\mathrm{solv}}^{S1\to S2}-\Delta G_{\mathrm{solv}}^{S1}=0$$

Rearranging eq 1, one sees immediately that

2 $$\Delta \Delta G_{\mathrm{solv}}=\Delta G_{\mathrm{solv}}^{S2}-\Delta G_{\mathrm{solv}}^{S1}=\Delta G_{\mathrm{solv}}^{S1\to S2}-\Delta G_{\mathrm{gas}}^{S1\to S2}$$

In other words, rather than taking the difference between the two physical paths (vertical arrows), we take the difference between the two horizontal paths in Figure 1. These involve nonphysical transformations that we refer to as *alchemical* paths. Provided all transformations can be computed independently, the requirement of cycle closure additionally offers a powerful internal consistency check, since the individual free energy contributions must sum to zero within statistical uncertainty (eq 1). Analogous thermodynamic cycles exist for other applications, e.g., binding free energy differences or the calculation of partition coefficients.^3^

It should be kept in mind that the technical challenges of realizing a transformation along an alchemical $\left(\Delta G_{\mathrm{gas}}^{S1\to S2},\;\Delta G_{\mathrm{solv}}^{S1\to S2}\right)$ and chemical path $\left(\Delta G_{\mathrm{solv}}^{S1},\;\Delta G_{\mathrm{solv}}^{S2}\right)$ are different: For chemical paths, one has to deal with the so-called van der Waals end point problem.^47^ In force field based FES, one employs so-called soft-core potentials^48–50^ though other approaches exist.^51^ As already mentioned in the Introduction, several methods for MLIP based FES have also been proposed.^39–42^ By contrast, the primary challenge arising along alchemical paths is that most of the time the number of atoms is different in the initial and final state. When using force fields, this can be handled, e.g., by the use of so-called dummy atoms.^52^ To the best of our knowledge, there does not exist a generally applicable approach to achieve such transformations in MLIP based FES.

### Distance Dependence in MLIPs

A central challenge in the design of MLIPs is the construction of a physically meaningful representation of a chemical system that remains invariant with respect to system size. To this end, most modern MLIP architectures decompose the total potential energy into a sum of atomic energy contributions, which depend on the local chemical environment of each atom. This atom-wise decomposition allows the model to be applied to systems containing arbitrary numbers of particles.

The majority of MLIP architectures, including MACE, is based on graph neural networks, which learn a representation vector for each atom from its local environment. Another approach is to construct atomic environment vectors, as used, for example, in the ANI architecture.^15,16^ In both cases, these atom-wise representations are subsequently used to predict atom-wise contributions to the total energy. To limit computational cost and ensure locality, interactions are restricted to atoms within a finite cutoff radius *r*_cut_, analogous to the use of cutoffs in classical force fields. That is, for each atom *i* = 1, ..., *N* in a system containing *N* particles, a representation vector *h*_*i*_ is constructed from its local neighborhood:

$$N\left(i\right)=\left\{j\neq i\;|\;d_{ij}\leq r_{\mathrm{cut}}\right\}$$

where *d*_*ij*_ denotes the distance between atoms *i* and *j*. Contributions from neighboring atoms are smoothly turned off as their interatomic distances approach the cutoff, ensuring continuous and differentiable potential energy surfaces. As a consequence, the influence of one atom on another decreases continuously with increasing distance. Both the atomic energy contributions and the representations that underlie them depend explicitly on these distance-weighted interactions. Manipulating interatomic distances—or, equivalently, modulating whether specific neighbors contribute to an atom’s local environment—therefore provides a natural mechanism for scaling interactions within an MLIP. In principle, a controlled reduction of such distance-dependent contributions can be used to smoothly attenuate interactions, a property that can be exploited for alchemical transformations without modifying the underlying neural network architecture.

### Manipulating Interactions by a Dual-Coordinate Approach

We introduce a method for computing relative free energy differences between two arbitrary solutes in aqueous solution based on a *dual-coordinate* approach[Fn ba-fn1]. In this framework, both solutes are present simultaneously and explicitly throughout the simulation. No atoms are created or removed during the transformation; instead, only their interactions with the surrounding environment are modulated. The interactions between solute atoms and the remainder of the system are controlled by a coupling parameter λ. At λ = 0, the first solute (denoted as *S*1) interacts fully with the solvent, while the second solute (denoted as *S*2) is completely decoupled from all interactions with its environment. As λ is increased, the interactions between *S*1 and the surrounding water molecules are gradually turned off, while, simultaneously, the interactions between *S*2 and the solvent are smoothly turned on. At λ = 1, *S*1 is fully decoupled from the environment and *S*2 interacts fully with the solvent. Thus, at both end points of the transformation (λ = 0 and λ = 1), the system consists of a fully interacting solute–water system coexisting with a second solute that effectively resides in the gas phase (see Figure 2). Details concerning the validation of the correct end point coupling/decoupling are provided in the Supporting Information. Importantly, intramolecular interactions within each solute are never modified. Consequently, both solutes retain their full internal structure and dynamics at all values of λ. In addition, it is essential that the two solutes do not interact with each other at any point during the simulation. Accordingly, all direct interactions between the two solutes are excluded for all values of the coupling parameter λ.

**Figure 2:**
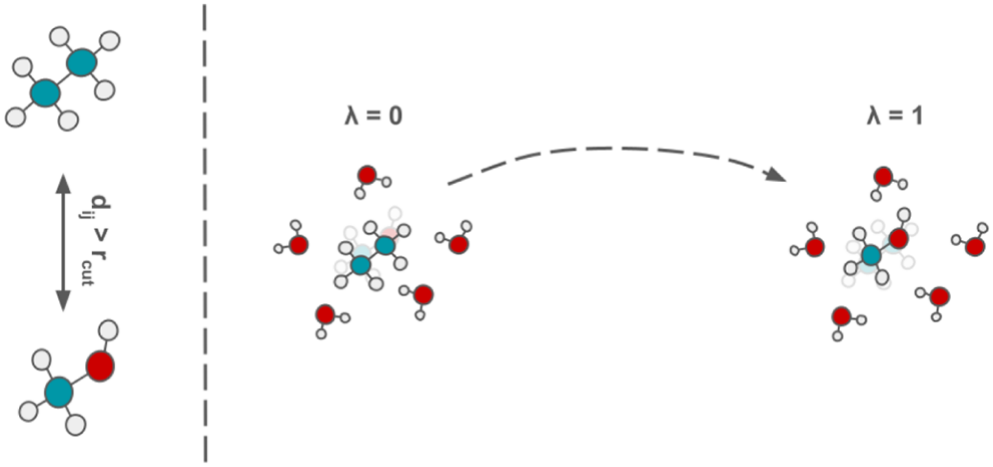
Solutes are masked from each other at all times using a constant distance offset, ensuring *d_ij_* > *r*_cut_. Interactions with surrounding water molecules are turned off for one solute (here ethane) and simultaneously turned on for the other solute (here methanol). At both end points of the transformation (λ = 0 and λ = 1), the system consists of a fully interacting solute–water system coexisting with a second solute that effectively resides in the gas phase.

For each intermediate state 0 < λ < 1, the solvent molecules partially interact with *S*1 and *S*2 simultaneously. Thus, this simultaneous decoupling-coupling procedure enables a direct alchemical transformation between the two solutes by manipulating solute–solvent interactions only. The solutes themselves are never modified in an unphysical manner but retain a "chemical topology''[Fn ba-fn1].

Analogous to our previous work on ASFEs,^42^ we employ a 4D-decoupling strategy to modulate interactions between solutes and solvent molecules. In contrast to strict absolute transformations, relative free energy calculations require a combination of three distinct distance-manipulation schemes that are applied simultaneously: (i) decoupling *S*1 from the solvent, (ii) coupling *S*2 to the solvent, and (iii) permanently decoupling *S*1 and *S*2 from one another.

The first component corresponds directly to the solute–solvent decoupling strategy introduced in ref 42. Starting from a fully interacting system, all distances between atoms of *S*1 and surrounding water molecules are modified by adding a λ-dependent offset. Based on previous benchmarks (see ref 42), we adopt a linear-to-cutoff scheme: For each λ ∈ [0,1], all interatomic distances $d_{ij}^{S1,\mathrm{solv}}\left(\lambda \right)$ between a solvent and an *S*1 atom are manipulated by adding a λ-dependent offset:

$$d_{ij}^{S1,\mathrm{solv}}\left(\lambda \right)=d_{ij}+\lambda \times \left(\max\left(0,r_{\mathrm{cut}}-d_{ij}\right)+\epsilon \right)$$

At λ = 0, $d_{ij}^{S1,\mathrm{solv}}$ is unmodified; hence, *S*1 interacts fully with the solvent, whereas at λ = 1, all *S*1–solvent interactions are effectively switched off by shifting the corresponding distances to the cutoff radius (plus ϵ > 0, to account for rounding errors, chosen based on the machine precision used: 10^–6^ for single precision and 10^–9^ for double precision). The second component, coupling *S*2 to the solvent, is implemented by reversing the decoupling protocol. Instead of turning interactions off, they are gradually introduced using the complementary λ dependence:

$$d_{ij}^{S2,\mathrm{solv}}\left(\lambda \right)=d_{ij}+\left(1-\lambda \right)\times \left(\max\left(0,r_{\mathrm{cut}}-d_{ij}\right)+\epsilon \right)$$

Consequently, at λ = 0, *S*2 is completely decoupled from the solvent, while at λ = 1 it fully interacts with the surrounding water molecules. To ensure that the two solutes never interact with each other, we apply a permanent distance offset to all *S*1–*S*2 atom pairs that is independent of λ:

$$d_{ij}^{S1,S2}\left(\lambda \right)=d_{ij}+\max\left(0,r_{\mathrm{cut}}-d_{ij}\right)+\epsilon \geq r_{\mathrm{cut}}+\epsilon$$

This guarantees that $d_{ij}^{S1,S2}\left(\lambda \right)>r_{\mathrm{cut}}$ at all times, thereby excluding any direct interactions between the two solutes (see Figure 2). All other interatomic distances remain unchanged. For convenience, we define a general distance-manipulation function

$$d_{ij}\left(\phi \right)=d_{ij}+\phi \times \left(\max\left(0,r_{\mathrm{cut}}-d_{ij}\right)+\epsilon \right)$$

which allows all cases to be expressed compactly as

3 $$d_{ij}^{u,v}\left(\lambda \right)=\left\{ \begin{array}{lll} d_{ij}\left(\lambda \right), & \left(u,v\right)=\left(S1,\;\mathrm{solv}\right) & \mathrm{case}\left(a\right),\\ d_{ij}\left(1-\lambda \right), & \left(u,v\right)=\left(S2,\;\mathrm{solv}\right) & \mathrm{case}\left(b\right),\\ d_{ij}\left(1\right), & \left(u,v\right)=\left(S1,\;S2\right) & \mathrm{case}\left(c\right),\\ d_{ij}\left(0\right), & \mathrm{else} & \mathrm{case}\left(d\right) \end{array}\right.$$

Importantly, at no point are the Cartesian coordinates of the atoms modified. Only the distance information that is passed to the MLIP is altered. In other words, even though a constant offset is applied to all *S*1–*S*2 atom pairs at the level of the MLIP input, this does not imply that the two solutes are being separated in space, which, in fact would be undesirable (see below). Because the MLIP propagates the manipulated distance information, no repulsive interactions arise between solute atoms, allowing both solutes to occupy the same region of space.

### Anchor

In the absence of additional restraints, the two solutes can and will diffuse freely throughout the simulation box, as they do not share a common core or subset of atoms. However, to carry out the alchemical transformation efficiently, it is desirable to keep the two solutes spatially close and approximately superimposed. This requires (1) the introduction of additional restraint potentials that serve to anchor both solutes at nearly the same position in the simulation box, and (2) carefully prepared initial coordinates and simulation box configuration; for the practical aspects, see "Starting Coordinates and Anchor'' in Methods.

Spatial overlap of the two solutes can be enforced by applying distance restraints between pairs of atoms, one in *S*1 and the other in *S*2; see, e.g., refs 53 or 54. However, care must be taken to ensure that the presence of the restraints does not affect the structure and dynamics of the physical solutes.^52,55^ In this work, we, therefore, select a *single* reference atom for each of the two solutes, denoted as anchor atom, typically located near the molecular center. We then define two anchor points in the simulation box, one for each solute, chosen to be very close to one another. These anchor points are only determined by 3D coordinates, there are no additional particles required. A harmonic positional restraint is applied between each selected anchor atom and its corresponding anchor point, implemented as a simple quadratic potential.

Let *X* = (*x*,*y*,*z*)^*T*^ denote the Cartesian coordinates of the chosen anchor atom of the solute and let $r_{\mathrm{anchor}}\in R^{3}$ denote the Cartesian coordinates of the anchor point. We set an equilibrium distance to the chosen anchor point. This equilibrium distance will be denoted as $r_{0}\in R$ . The distance vector between the anchor point *r*_anchor_ and the anchor atom *X* will be denoted as $\mathrm{r⃗}=X-r_{\mathrm{anchor}}\in R^{3}$ . The position restraint is defined as

4 $$F_{\mathrm{custom}}=0.5\cdot k\cdot {\left(|\mathrm{r⃗}|-r_{0}\right)}^{2}$$

with *k* being the force constant. That is, we allow the anchored atom to move on the surface of a sphere with radius *r*_0_ around the anchor point *r*_anchor_ (see Figure 3).

**Figure 3:**
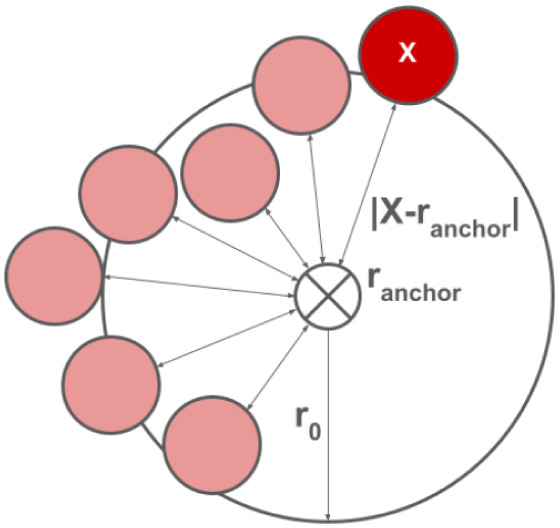
2D projection of anchored atom moving on the surface of a sphere around the anchor point.

An application of two such harmonic restraints ensures that core parts of the two solutes are restrained to occupy the same spatial location throughout the simulation. Importantly, because all intramolecular interactions remain unmodified, each solute retains its full structural integrity, and the applied restraints restrict the movements of the entire molecules rather than isolated atoms. The restraint potentials are independent of the coupling parameter λ and are applied with identical force constants for both solutes at all stages of the transformation. Consequently, these additional force terms contribute equally to all thermodynamic states and cancel in the calculation of relative free energy differences. Additional details on the statistical derivation of solvation free energy differences and their independence from position and orientation of the solute—hence anchor restraints do not influence the computation of solvation free energy differences—are given in the Supporting Information.

Our dual-coordinate approach in combination with restraints as just described bears some resemblance to the Separated Topologies (SepTop) method used in alchemical FES with classical force fields to determine binding free energy differences.^56^ In the SepTop approach, one executes two absolute binding free energy calculations simultaneously in opposite directions, one for each ligand. In other words, ref 56 also modifies the interactions between distinct molecules and their environment rather than directly transforming the molecules themselves. During the alchemical transformation for the protein–ligand complex, a set of six restraints restricting the position and orientation of the ligands relative to the protein are used.

### Validation by Cycle Closure

MLIPs, including the MACE-OFF23­(S) potential used in this work, are trained on data sets consisting mostly of single-point energies, making their use in dynamic simulations an application far outside their training domain. Consequently, they do not always reproduce meaningful condensed-phase properties^32,57^ that are critical for free energy simulations. Agreement of the calculated RSFEs with experiment, therefore, may not be a reliable indicator of correctness. Therefore, we assess the reliability of our approach by examining thermodynamic cycle closure, combining relative and absolute solvation free energy differences between solute pairs.

When discussing chemical and alchemical paths in the Theory section (Figure 1), we already provided the cycle closure criterion eq 1 and the resulting eq 2. Since we are using a dual-coordinate approach, combined with restraints that do not contribute to the relevant free energy differences, eq 2 can be simplified further. Specifically, both solutes are present in all intermediate λ states, they never interact with each other, and they retain their full intramolecular structures (see also Figure 2). Therefore, the relative gas-phase contribution vanishes, i.e.,

$$\Delta G_{\mathrm{gas}}^{S_{1}\to S_{2}}=0$$

Therefore, eq 2 reduces to

5 $$\Delta G_{\mathrm{solv}}^{S_{1}\to S_{2}}=\Delta G_{\mathrm{solv}}^{S_{2}}-\Delta G_{\mathrm{solv}}^{S_{1}}$$

We use eq 5 as a straightforward means to validate our method: by combining ASFEs with the RSFE differences computed via the present alchemical transformation protocol, we can test the internal consistency of the approach and confirm the correctness of the computed relative free energies.

## Methods

### Choice of Solutes and Solute Pairs

#### Small Solutes

Our approach is a direct extension of the methodology presented and tested in ref 42. Therefore, we consider all model solutes from this study and reuse the ASFE results reported there whenever possible. Inspired by ref 58, we extend the set of seven solutes from ref 42—water, methane, methanol, toluene, phenol, ethane, and ethanol—by four additional molecules: methylthiol, acetone, acetamide, and tetrahydrofuran (see Figure 4). The König et al. set^58^ also includes hexane and cyclohexane, but since these two molecules might require extensive simulation lengths to converge because of their internal flexibility, we do not consider them in this work.

**Figure 4:**
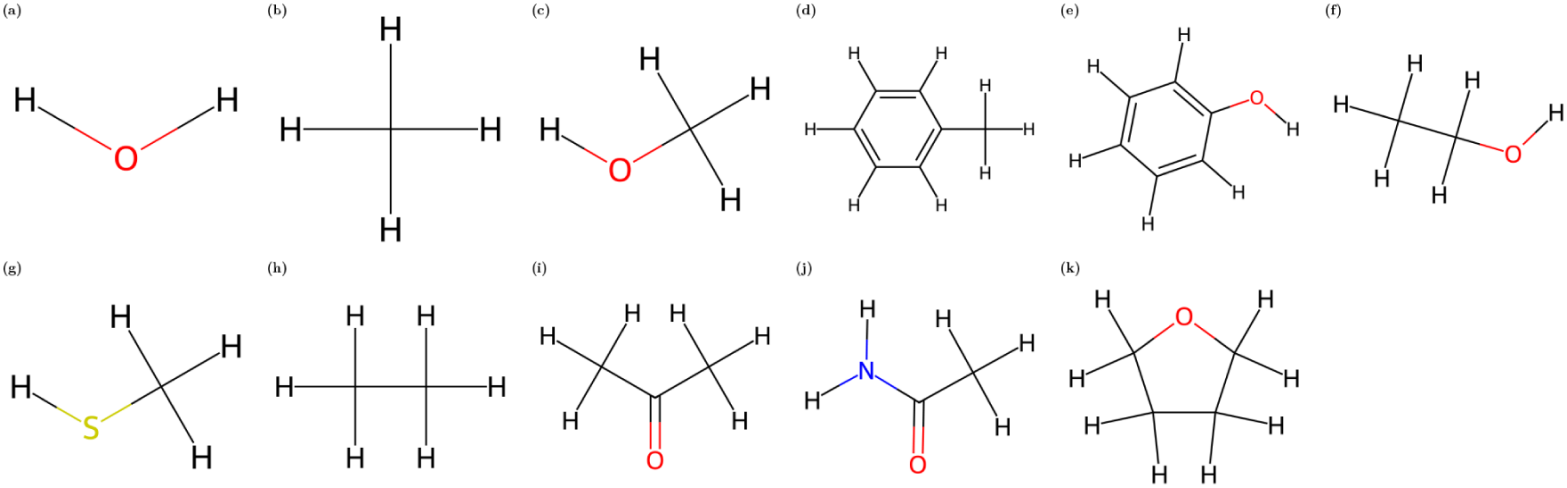
Solutes for ASFE calculations: (a) Water, (b) Methane, (c) Methanol, (d) Toluene, (e) Phenol, (f) Ethanol, (g) Methylthiol, (h) Ethane, (i) Acetone, (j) Acetamide, and (k) Tetrahydrofuran.

#### Solute Pairs

A dual-coordinate approach makes it straightforward to compute the RSFE between any two solutes; i.e., there is no need for chemical similarity. Given 11 solutes, we could, in principle, compute 55 RSFEs. To limit the computational cost, we calculated RSFEs only between selected pairs of solutes, *S*1 and *S*2. We tried to cover a diverse set of alchemical transformations, such as change in polarity and change in solute size. We also include transformations between very similar solutes, for which the RSFE is small.

### Starting Coordinates and Anchor

#### Creating Starting Coordinates for a Solute Pair *S*1 → *S*2

For both solutes *S*1 and *S*2, we first separately equilibrated the corresponding solvated systems using simulation boxes of similar size. Subsequently, all coordinates were shifted such that each solute was centered in its respective box, and all solvent molecules were removed from the equilibrated and recentered *S*2 system. The coordinates of *S*2 were then superimposed onto those of *S*1 in its corresponding solvated and equilibrated box, which still contains all water molecules (see Figure 5). To avoid initial overlap with solvent molecules, we always removed water molecules from the box containing the smaller solute (i.e., the ordering *S*1 → *S*2 was chosen such that *S*1 is larger than *S*2).

**Figure 5:**
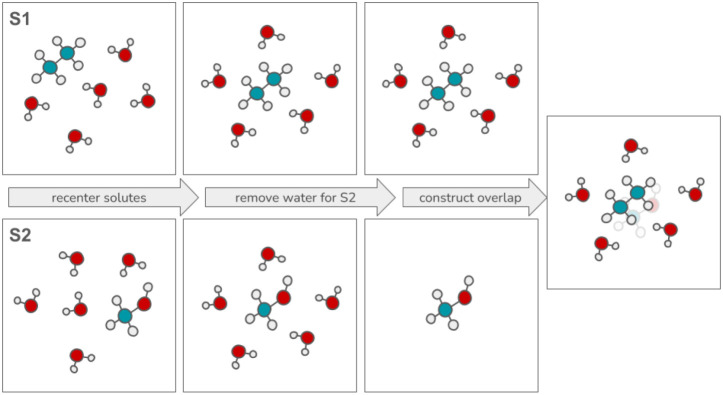
From left to right: Start with two separately equilibrated boxes for both solutes *S*1 (here ethane) and *S*2 (here methanol). Next, recenter both solutes in their corresponding boxes. Remove all solvent molecules from the *S*2 box. Finally, place *S*2 on top of *S*1 in the *S*1-solvated box by aligning the anchored atoms and the helper orientation axes.

The two solutes were aligned as follows: in addition to the anchor atom (corresponding to the anchor atom used for the position restraint), we selected one helper atom to define an orientation axis. The axis defined by these two atoms was computed for both solutes. *S*2 was then placed such that the anchor atoms nearly coincide and the corresponding axes are aligned. In practice, a small initial separation (0.05 Å) between the two anchor atoms is retained to avoid numerical issues.

#### Anchor Restraints

We used CustomExternalForce from the OpenMM library^59^ to apply the harmonic position restraints. This allows us to introduce a custom-tailored quadratic restraint term that can be applied to arbitrary particles in the system (see eq 4). We use two anchor points, one for each of the two solutes. The anchor point positions, i.e., the 3D coordinates of $r_{\mathrm{anchor}}^{S1}$ and $r_{\mathrm{anchor}}^{S2}$ used to define *r*_anchor_ in eq 4, were set to the initial coordinates of the corresponding anchor atoms after starting coordinates were created (see above). Hence, the anchor points almost coincide. In this work, we used a force constant *k* = 100 kcal/mol/Å^2^ for both anchors and all solute pairs. To avoid overly restraining the solute motion, the equilibrium distance *r*_0_ was always set to *r*_0_ = 0.05 Å.

### Simulation Details and Free Energy Calculations

#### Simulation Details

To maintain consistency with the results obtained in ref 42, all simulations in the present work closely followed the setup and protocols reported there. The average size of the simulation box in all cases was ≈23 Å. Details regarding the number of water molecules for each solute and the corresponding average box dimensions are provided in the Supporting Information, Table S6.

All simulations were carried out with the OpenMM-ML framework[Fn ba-fn2],^59^ using the MACE-OFF23­(S) model.^24^ All simulations were run under constant pressure and temperature conditions for 1 ns using the Langevin integrator and a time step of 1 fs. The pressure was kept constant by applying a Monte Carlo barostat.^60,61^ Coordinates were saved every 0.25 ps, and the initial 20% of each simulation was discarded to allow for equilibration. Only every fourth of the saved coordinate sets, i.e., 800 configurations per λ-state, were used for the actual free energy calculation. In rare cases, simulations exhibited instabilities when using a 1 fs time step (observed in only 4 out of 660 simulations, corresponding to ≈0.6%); such systems were rerun using a reduced integration time step of 0.5 fs, which resulted in stable trajectories. Saving frequencies, etc., were doubled in these cases.

#### Calculation of Free Energy Differences

ASFE differences, $\Delta G_{\mathrm{solv}}^{S1},\;\Delta G_{\mathrm{solv}}^{S2}$ , and RSFE differences, $\Delta G_{\mathrm{solv}}^{S1\to S2}$ , were computed from equilibrium simulations at *N*_λ_ discrete values of the coupling parameter λ (see below for details). We typically employ the MBAR algorithm to calculate free energy differences,^62^ specifically the pymbar implementation[Fn ba-fn3]. However, as already observed in ref 42, when applied to data generated with the 4D-decoupling approach to compute ASFEs, pymbar often failed to converge. As discussed in detail in ref 42, this behavior originates from configurations in which atoms approach each other very closely and the MLIP falsely predicts excessively negative potential energies. These configurations consequently receive disproportionately large statistical weights, which impairs the stability of the estimator. For this reason, as in ref 42, we instead employed the BAR estimator as implemented in pymbar to compute the free energy differences between neighboring λ-states. The total free energy difference is then obtained as a sum of pairwise contributions along the alchemical path,

$$\Delta G=\overset{N_{\lambda }}{\underset{i=1}{\sum }}\Delta G_{\lambda_{i}}$$

where each pairwise free energy difference $\Delta G_{\lambda_{i}}$ is estimated from simulations performed at the two neighboring coupling states λ_*i*_ and λ_*i*+1_.

For the computation of ASFEs, we initially employed a set of 20 equidistant λ-states. As described in ref 42, the uncertainties of the pairwise free energy contributions assigned by the BAR algorithm were negligible for λ > 0.5. This observation allowed for the removal of several λ-points in that region, reducing the total number of states used to 15. All additional ASFE calculations carried out in this work used this protocol. By contrast, for the relative free energy calculations presented here, a symmetric λ-schedule is needed because decoupling of solute *S*1 and (re)­coupling of solute *S*2 occur simultaneously, requiring a denser and symmetric spacing of λ-states during the second half of the transformation (λ > 0.5). Accordingly, all 20 λ-states were retained for the relative calculations. That is, for each alchemical transformation, we used 20 equidistant λ-states,

$$\lambda \in \left\{\frac{i}{19}|i=0,...,19\right\}$$

We did not attempt to optimize this set of λ-windows; such an optimization would likely require system-specific tuning of the λ distribution. To demonstrate that the chosen λ-schedule nonetheless provides sufficient overlap between neighboring states, we present two representative pairwise overlap matrices in the Supporting Information.

Each absolute or relative free energy difference Δ*G* was computed as the mean of three repeats started from different initial random velocities; the standard deviation of the three individual Δ*G* results was used as the error estimate. Calculations were carried out on NVIDIA RTX4090 cards; on this hardware, 1 ns of simulation with a 1 fs time step required approximately 9 h of wall-time. We report results for both the ASFEs for each of the small solutes and for the RSFEs for each of the solute pairs studied.

## Results

### Absolute Solvation Free Energies

ASFEs for the 11 solutes are tabulated in Table 1 and the results are depicted graphically in Figure 6. Overall, the calculated ASFEs, $\Delta G_{\mathrm{solv}}^{\mathrm{calc}}$ , agree well with the experimental data $\Delta G_{\mathrm{solv}}^{\exp}$ . The hydration affinity of the model compounds ranges from highly hydrophobic (methane, ethane, $\Delta G_{\mathrm{solv}}^{\exp}\approx +2.0\;\mathrm{kcal}/\mathrm{mol}$ ) to highly hydrophilic (acetamide, $\Delta G_{\mathrm{solv}}^{\exp}\approx -10.0\;\mathrm{kcal}/\mathrm{mol}$ ). By far the largest deviation is observed for methylthiol $\left(\epsilon \left(\Delta G_{\mathrm{solv}}\right)=\Delta G_{\mathrm{solv}}^{\mathrm{calc}}-\Delta G_{\mathrm{solv}}^{\exp}=1.48\;\mathrm{kcal}/\mathrm{mol}\right)$ , the only solute in the test set containing a sulfur atom. For all other solutes, |ϵ­(Δ*G*_solv_)| remains below 1 kcal/mol. The statistical uncertainties of all calculations are low, 0.03–0.18 kcal/mol, a magnitude consistent with what one would expect for force-field-based ASFE calculations for small solutes, using comparable simulation protocols.

**Table 1:** Calculated $\Delta G_{\mathrm{solv}}^{\mathrm{calc}}$ and Experimental Solvation Free Energies $\Delta G_{\mathrm{solv}}^{\exp}$ in kcal/mol^a^

Solute	$\Delta G_{\mathrm{solv}}^{\mathrm{calc}}$	$\Delta G_{\mathrm{solv}}^{\exp}$	ϵ(Δ*G*_solv_)
Water*	–6.48 ± 0.05	–6.30	–0.18
Methane*	+2.03 ± 0.03	2.00	0.03
Ethane*	+2.26 ± 0.13	1.83	0.43
Methanol*	–4.79 ± 0.10	–5.10	0.31
Ethanol*	–4.25 ± 0.12	–5.00	0.75
Toluene*	–1.26 ± 0.07	–0.90	–0.36
Phenol*	–5.96 ± 0.13	–6.60	0.64
Acetone	–3.03 ± 0.17	–3.80	0.77
Acetamide	–9.44 ± 0.15	–9.71	0.27
Tetrahydrofuran	–3.24 ± 0.18	–3.47	0.23
Methylthiol	+0.28 ± 0.11	–1.20	1.48

a The experimental values are taken from the FreeSolv^7^ or the Minnesota solvation database.^67^ A star (*) next to the molecule name indicates that the $\Delta G_{\mathrm{solv}}^{\mathrm{calc}}$ result is from ref 42. $\epsilon \left(\Delta G_{\mathrm{solv}}\right)=\Delta G_{\mathrm{solv}}^{\mathrm{calc}}-\Delta G_{\mathrm{solv}}^{\exp}$ .

**Figure 6:**
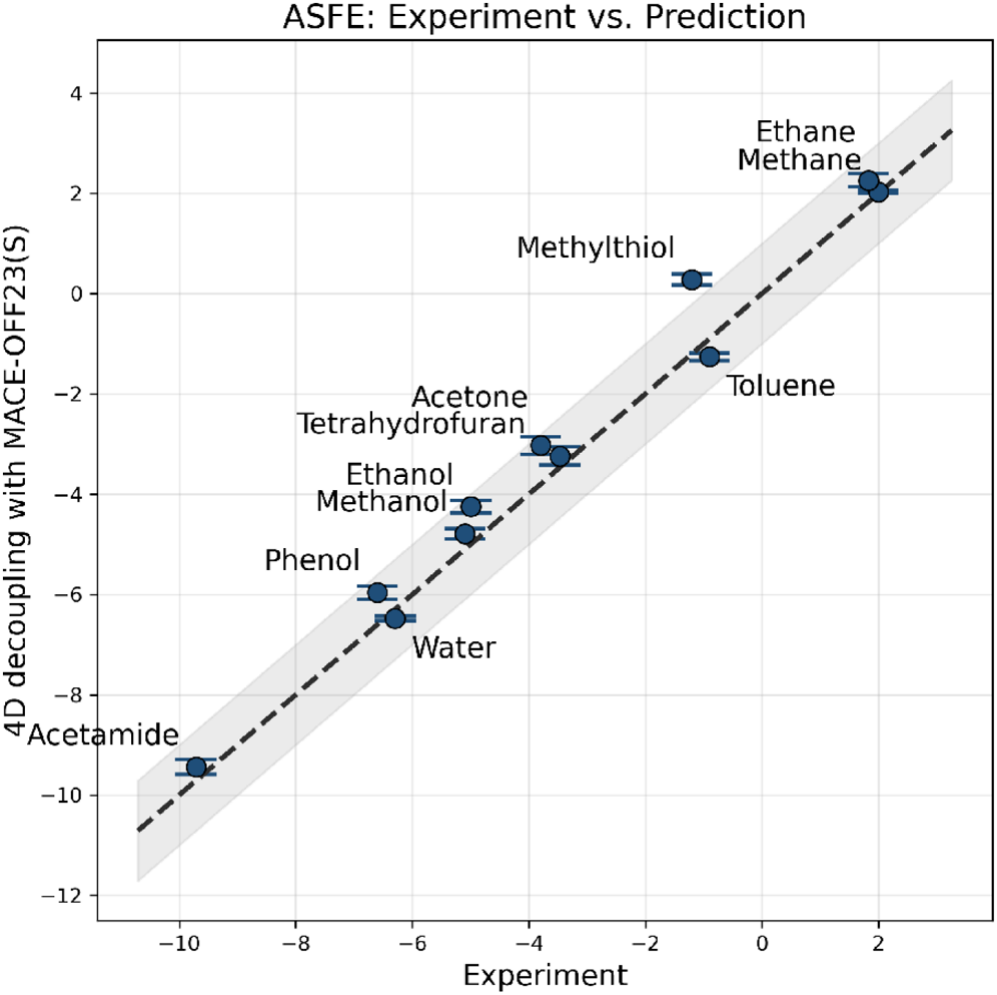
Scatter plot of $\Delta G_{\mathrm{solv}}^{\exp}$ (*x*-axis) vs $\Delta G_{\mathrm{solv}}^{\mathrm{calc}}$ (*y*-axis), all values in kcal/mol. The dashed line represents the identity line (*y* = *x*), and the gray-shaded region indicates deviations of ±1 kcal/mol from the identity.

With the exception of water, all ASFEs reported here have been computed multiple times in systematic, large-scale studies using force fields. Specifically, we compare to the results obtained by Karwounopoulos et al.^8^ and He et al.^9^ (see Table S7 in the Supporting Information). For the 10 solutes considered here (all except water), the 4D-decoupling approach combined with the MACE-OFF23­(S) potential yields a mean absolute error (MAE) of 0.45 kcal/mol and a root-mean-square-deviation (RMSD) of 0.66 kcal/mol. For the same solutes, the results of Karwounopoulos et al., using the CGenFF force field,^63–66^ yielded MAE/RMSD values of 0.83/1.11 kcal/mol. The MAE/RMSD values obtained with the AMBER GAFF force field and the new ABCG2 charge assignment scheme^9^ were 0.41/0.45 kcal/mol. Although the number of solutes is too small to draw definitive conclusions, it is encouraging that FES using an MLIP gives results consistent with established classical force field methods. To contextualize the MAE/RMSD values, one should keep in mind that the ABCG2 charge model^9^ was developed with a clear focus on solvation free energies.

### Relative Solvation Free Energies

The RSFE results $\Delta G_{\mathrm{solv}}^{S1\to S2}$ for all solute pairs obtained with the dual-coordinate approach in combination with anchoring are summarized in Table 2. The direct RSFE results are compared with $\Delta \Delta G_{\mathrm{solv},4D}^{S1,S2}=\Delta G_{\mathrm{solv}}^{S2}-\Delta G_{\mathrm{solv}}^{S1}$ , the difference of the two individual ASFE results (see the right-hand side of eq 5). $\Delta \Delta G_{\mathrm{solv},4D}^{S1,S2}$ was computed as the difference between the respective means of the two ASFEs (see Table 1), and the associated standard deviation was obtained via Gaussian error propagation from the ASFE standard deviations. The cycle closure error, presented in the rightmost column in Table 2, is calculated as $\Delta \Delta G_{\mathrm{solv},4D}^{S1,S2}-\Delta G_{\mathrm{solv}}^{S1\to S2}$ and should be zero within statistical error bars. The mean and standard deviation of the cycle closure error were calculated as the difference between the mean values of $\Delta \Delta G_{\mathrm{solv},4D}^{S1,S2}$ and $\Delta G_{\mathrm{solv}}^{S1\to S2}$ , with uncertainties again estimated using Gaussian error propagation.

**Table 2:** Summary of RSFE Results $\left(\Delta G_{\mathrm{solv}}^{S1\to S2}\right)$ and Cycle Closure $\left(\Delta \Delta G_{\mathrm{solv},4D}^{S1,S2}-\Delta G_{\mathrm{solv}}^{S1\to S2}\right)\;\mathrm{in}\;\mathrm{kcal}/\mathrm{mol}$ a

*S*1	*S*2	$\Delta \Delta G_{\mathrm{solv},\exp}^{S1,S2}$	$\Delta G_{\mathrm{solv}}^{S1\to S2}$	$\Delta \Delta G_{\mathrm{solv},4D}^{S1,S2}$	Cycle closure
Ethane	Water	–8.13	–8.85 ± 0.19	–8.74 ± 0.14	+0.11 ± 0.24
Water	Ethane	8.13	+8.76 ± 0.17	+8.74 ± 0.14	–0.02 ± 0.21
Ethane	Methanol	–6.93	–6.94 ± 0.26	–7.05 ± 0.16	–0.11 ± 0.31
Toluene	Phenol	–5.70	–4.68 ± 0.14	–4.69 ± 0.15	–0.01 ± 0.21
Ethanol	Methane	7.00	+6.35 ± 0.24	+6.29 ± 0.12	–0.06 ± 0.27
Phenol	Ethane	8.43	+8.18 ± 0.03	+8.21 ± 0.18	+0.03 ± 0.18
Methane	Ethane	–0.17	+0.20 ± 0.12	+0.22 ± 0.13	+0.02 ± 0.17
Methanol	Ethanol	0.10	+0.50 ± 0.31	+0.53 ± 0.15	+0.03 ± 0.34
Methylthiol	Ethane	3.03	+2.02 ± 0.07	+1.97 ± 0.17	–0.05 ± 0.18
Acetone	Acetamide	–5.91	–6.37 ± 0.29	–6.41 ± 0.23	–0.04 ± 0.37
Toluene	Tetrahydrofuran	–2.57	–2.07 ± 0.12	–1.98 ± 0.20	+0.09 ± 0.23

a $\Delta \Delta G_{\mathrm{solv},4D}^{S1,S2}=\Delta G_{\mathrm{solv}}^{S2}-\Delta G_{\mathrm{solv}}^{S1}$ , with the ASFE values, $\Delta G_{\mathrm{solv}}^{S1}$ and $\Delta G_{\mathrm{solv}}^{S2}$ , taken from Table 1. $\Delta G_{\mathrm{solv}}^{S1\to S2}$ is the result obtained from the direct alchemical dual-coordinate transformation. For ease of comparison, we also report experimental values for the relative solvation free energies $\Delta \Delta G_{\mathrm{solv},\exp}^{S1,S2}$ .

First, we investigated whether the slight “asymmetry” in the setup of the simulation systems used for the alchemical transformations (cf. Methods) influences the results. Normally, each simulation is started from equilibrated coordinates obtained for the system containing the larger solute, with the smaller solute superimposed as described in the Methods section. The first two entries in Table 2 report RSFEs for the solute pair water–ethane, for which we carried out the calculations once using equilibrated coordinates for ethane in water, and once starting all simulations from equilibrated coordinates for water in water, with the larger ethane superimposed. As one sees in Table 2, the two $\Delta G_{\mathrm{solv}}^{S1\to S2}$ results agree very well, −8.85 ± 0.19 vs +8.76 ± 0.17 kcal/mol, indicating that the setup of the simulations has no influence on the result. Furthermore, both series of simulations were equally stable, even though in the water → ethane direction the larger ethane is “grown” from the smaller water molecule. The small cycle closure error for the two directions also lies within the statistical error bars.

Turning next to all other RSFEs, which were only computed in one direction, all mean cycle closure errors reported in Table 2 lie within the range [−0.11, 0.09] kcal/mol and can, therefore, be considered statistically indistinguishable from zero, given the associated uncertainties of 0.17–0.37 kcal/mol. These observed low statistical uncertainties for all RSFEs are consistent with expectations for force-field-based calculations performed with a comparable protocol (three independent repeats and simulation times of <1 ns per λ-state).

As mentioned, the 11 model compounds range from very hydrophobic to very hydrophilic, and the chosen solute pairs combine various types of molecules. In addition to ethane–water, we also computed the RSFE for ethane–methanol, the first example of an alchemical transformation, studied as early as 1985.^68^ Several additional pairs also involve transformations between a hydrophobic and a hydrophilic solute (toluene–phenol, ethanol–methane, phenol–ethane). The results demonstrate that our RSFE methodology handles all transitions between apolar and polar well, regardless of whether the solutes have similar (toluene–phenol) or different sizes (phenol–ethane). The pairs methane–ethane and methanol–ethanol are examples of two hydrophobic and two hydrophilic solutes. The (absolute value of the) RSFE for each pair is small; yet, the $\Delta G_{\mathrm{solv}}^{S1\to S2}$ and $\Delta G_{\mathrm{solv},4D}^{S1,S2}$ values agree excellently, i.e., the cycle closure error is close to zero. Although the ASFE of methylthiol is in poor agreement with the experiment, the cycle closure error for methylthiol–ethane is low. The alchemical transformation of acetone to acetamide is one of the pairs with a relatively large standard deviation (0.37 kcal/mol). Even though the two solutes are of comparable size, this is a nontrivial change: not only is a tetrahedral methyl group replaced by a planar NH_2_ moiety, but the character of the bond also changes from a single to a (partial) double bond. The transformation of toluene to tetrahydrofuran (last entry in Table 2) involves a change in ring size. Such an alchemical change would be difficult using a single-coordinate approach (regardless of whether one uses “single topology” or “hybrid topology”), even if force fields were used. The low cycle closure error (+0.09 kcal/mol) and the low statistical uncertainty (0.23 kcal/mol) indicate that the dual-coordinate approach chosen is an efficient means to realize complicated alchemical transformations. Overall, the results demonstrate that the proposed method is robust with respect to the chemical nature of the chosen solute pair.

We emphasize that the cycle-closure validation presented here primarily serves as a consistency check to demonstrate that the simultaneous coupling/decoupling procedure is equivalent to the original 4D decoupling approach introduced in ref 42. We additionally checked cycle closure on a three-state thermodynamic cycle; these results are provided in the Supporting Information. We note that cycle closure was already demonstrated in ref 42, where an independent protocol, not using 4D-(de)­coupling for computing relative solvation free energies, was employed.

## Summary and Outlook

The dual-coordinate approach described in this work enables alchemical transformations between, in theory, arbitrary pairs of solutes. While the reasonable agreement of the calculated ASFEs using the MACE-OFF23­(S) potential with the experimental data is certainly encouraging, the negligible cycle closure errors are arguably the more important finding of this work. To the best of our knowledge, our dual-coordinate approach, in combination with suitable “anchors”, is the first generally applicable methodology to carry out relative alchemical transformations in systems fully described by MLIPs. The protocol presented here makes it possible to carry out alchemical transformations of small solutes in solution within a single simulation, i.e., along one set of λ states.

To date, only a limited number of approaches for relative transformations fully operating at the MLIP level have been reported,^43,44^ and these strategies typically come with limitations, such as requiring a high degree of chemical similarity between the solutes or necessitating custom MLIP training. In contrast, our dual-coordinate formulation does not require any minimum degree of chemical similarity and can use an existing foundation model without custom training. Mutations such as toluene to tetrahydrofuran—and to a lesser extent, acetone to acetamide—represent alchemical transformations that are trivial to set up in the dual-coordinate framework used in this work but significantly more complex using a single-coordinate approach. This inherent flexibility has prompted renewed interest in dual-coordinate methods even in FES using traditional force fields, exemplified by the SepTop method.^56^ The dual-coordinate approach also ensures computational efficiency by coupling and decoupling the two solutes from the system simultaneously. If a single-coordinate approach were used to morph one solute into the other, the nonadditive nature of MLIPs would necessitate two full evaluations of energies and forces.

The primary challenge of dual-coordinate approaches lies in defining restraints that effectively position the two independent molecules relative to each other. Even in force-field-based FES, no consensus exists on the optimal approach. e.g., while clamping endstates together via pairs of distance restraints^53,54^ is efficient, it can inadvertently perturb the internal structure and dynamics of the molecules.^52^ To avoid systematic errors from redundant restraint degrees of freedom, we employed a single anchor atom for each molecule. This restraint ensures the proximity of the two solutes without enforcing strict spatial overlap—an approach that appears sufficient for small, compact molecules. However, a single anchor is likely insufficient for larger solutes that sample extended, nonoverlapping regions of configurational space. Future work will therefore focus on more sophisticated restraint schemes to enforce consistent spatial overlap. Such refinements are expected to improve numerical stability and enable reliable free energy calculations for larger, flexible solutes, also paving the way for the computation of both absolute and relative binding free energies.

Our earlier work^42^ and the methodology presented here demonstrate that by straightforward manipulation of the neighbor list one can carry out free energy simulations along both chemical and alchemical paths. Since the neighbor list is central to most MLIP architectures, our approach can be extended to other MLIPs without requiring architecture-specific modifications. Thus, the identical methodology to carry out absolute and relative FES can be applied to a variety of MLIPs, facilitating the comparison of different MLIPs (e.g., with regard to network architecture and training data) to compute free energy differences of interest, e.g., ASFEs and RSFEs. The comparison of calculated free energy differences with experimental data thus can be used as a sensitive gauge of how well different MLIPs can reproduce a complex, central thermodynamic quantity.

## Supplementary Material



## Data Availability

A collection of the scripts that were used to compute the results presented here, as well as some input PDB files, can be found in the GitHub repository: https://github.com/cbc-univie/anchor-4D. Additionally, a frozen snapshot of the repository corresponding to the version used in this work has been archived on Zenodo (Anchor-4D repository: 10.5281/zenodo.19234256, forked OpenMM-ML repository: 10.5281/zenodo.19234287), with all code and data saved on March 26, 2026.

## Footnotes

1 See the ongoing work toward version 2.0 of ref 3 at https://github.com/alchemistry/alchemical-best-practices.

2
https://github.com/openmm/openmm-ml.

3
https://github.com/choderalab/pymbar.

## Notes

(i) Overview of MLIP methods for free energy computations, (ii) additional details on the independence of solvation free energies from anchor restraint(s), (iii) additional numerical consistency checks including endpoint validation, a three-state cycle closure and pairwise BAR overlap matrices for two solute pairs, (iv) additional numerical values including detailed information on the simulation systems including box size and number of solvent molecules, as well as numerical ASFE and RSFE results for all repeats, (v) additional details on the comparison to force field studies. (PDF)
