# Supplementary material for: Dual-Coordinate Relative Free Energy Simulations Using Machine-Learned Interatomic Potentials
Source: J Chem Theory Comput. 2026 Jul 22;22(15):7558–69. doi: 10.1021/acs.jctc.6c00824 (PMC13472084; doi:10.1021/acs.jctc.6c00824)
Supplement: Supplementary file 1 [file ct6c00824_si_001.pdf]

Supporting Information: Dual-Coordinate Relative Free Energy Simulations using Machine-Learned Interatomic Potentials

Anna Katharina Picha[*,†,‡] and Stefan Boresch[†]

†*University of Vienna, Faculty of Chemistry, Institute of Computational Biological Chemistry, 1090 Vienna, Austria*

‡*University of Vienna, Vienna Doctoral School of Chemistry (DosChem), 1090 Vienna, Austria*

E-mail: anna.katharina.picha@univie.ac.at

MLIP methods for free energy computations

We summarize several published methods for computing free energy differences with MLIPs and compare their key characteristics, requirements, and limitations in Table S1. The listed methods cover approaches for calculating ASFE, RSFE, absolute binding free energies (ABFE), and relative binding free energies (RBFE). We emphasize that our 4D-(de)coupling approach is the only method that requires only a single energy evaluation pass, does not require architectural modifications or additional training, does not require chemical similarity between solutes in RSFE calculations, and does not rely on a hybrid MM/ML framework. In principle, the method is also applicable to (1) fully MM-based systems and (2) ABFE and RBFE calculations. However, applications to binding free energy calculations are planned for future work and have not been tested yet.

Table S1: Overview of existing methods for free energy calculations with MLIPs.

Method	Approach	Used for	Remarks	Potential
Architectural modifications	Altered smoothing functions[a]<br>Altered message passing[b]	ASFE/ABFE<br>ASFE	Single energy pass	FENNIX[1]<br>MACE[2]
Hamiltonian Mixing	Single coordinate, dual topology[c]	RSFE	Double energy pass<br>Chemical similarity required	MACE-OFF23[3]
4D-(de)coupling	Pairwise distance offset[d]	ASFE<br>RSFE	Single energy pass<br>No chemical similarity required	MACE-OFF23[3]
Mixed Systems (MM/ML)	Buffer region[e]<br>Indirect approaches[f]<br>ML endstates, MM transformations[g]<br>ATM[h]	RSFE<br>ASFE/RBFE<br>ASFE<br>RBFE	Custom training<br>Mechanical embedding<br>No ML transformation<br>Large simulation systems	SchNet[4]/GROMOS[5,6]<br>ANI-2x[7]/CGenFF[8–10]<br>MPNICE[11]/LJ potential<br>ANI-2x[7]/GAFF2[12,13]<br>AceFF 1.0[i]/ AMBER[14,15]

[a] Ple et al. (Ref. 16)
[b] Moore et al. (Ref. 17)
[c] Tkaczyk et al. (Ref. 18)
[d] Picha et al. (Ref. 19)
[e] Crha et al. (Ref. 20)
[f] Karwounopoulos et al. (Ref. 21), Karwounopoulos et al. (Ref. 22)
[g] Xie et al. (Ref. 23)
[h] Sabanés Zariquiey et al. (Ref. 24), Sabanés Zariquiey et al. (Ref. 25)
[i] AceFF 1.0: https://huggingface.co/Acellera/AceFF-1.0

Independence of solvation free energies from anchor restraint(s)

Our use of anchor restraints may raise the concern that they influence relative solvation free energy differences. First, since the anchor restraint on each solute is always present, it does not depend on the coupling parameter λ and, therefore, makes no direct contribution to the calculated free energy differences. More importantly, the position and orientation of the solute have no influence on its solvation free energy. Since this aspect is easy to overlook in standard presentations, we briefly outline the statistical mechanical derivation of the solvation free energy, emphasizing its independence from the solute's location and orientation. For the full and more general presentation, see Ben-Naim and Marcus[26] and references cited therein.

The solvation free energy ΔA^0_{solv} of a solute A is defined as

$$\Delta A^0_{\text{solv}} = \mu_A^{0,l} - \mu_A^{0,g}, \tag{1}$$

i.e., as the difference between the standard chemical potential μ^0 of A in the liquid (l) and gas phase (g). For simplicity, we present our considerations in the canonical ensemble. At room temperature and pressure $\Delta A^0_{\text{solv}} \approx \Delta G^0_{\text{solv}}$. The gas phase is assumed to be ideal, and the solution ideally diluted, which in simulation settings (one solute only) is always guaranteed.

We start with the chemical potential of A in solution, which can be written as

$$\mu_A^l = -k_B T \ln \frac{V^l}{\Lambda_A^3} q_A^{l'} = -k_B T \ln \frac{1}{C^l\,\Lambda_A^3} q_A^{l'} \tag{2}$$

In Eq. 2, k_B is Boltzmann's constant and T the temperature. V^l is the volume of the simulation system, C^l is the corresponding number density $1/V^l$. Λ_A is the thermal de Broglie wavelength of the solute. The factor V^l/Λ_A^3 is the center of mass translational contribution to the partition function, the rest of the solute's partition function is denoted $q_A^{l'}$, with the prime $'$ indicating that the center of mass translational contribution has been factored out.

Therefore, $q_A^{l'}$ does *not* depend on the volume. Next, we express it in terms of the intramolecular configurational partition function z_A^l, i.e.,

$$q_A^{l'} = \frac{\Lambda_A^3}{\prod_i \lambda_i^3}\, 8\pi^2\, z_A^l \tag{3}$$

The λ_i, not to be confused with the coupling parameter, are the de Broglie wavelengths of each of the atoms of A; the leading fraction $\Lambda_A^3/\prod_i \lambda_i^3$ restores the separation of kinetic and potential energy contributions to the full partition function, possible in classical physics when cartesian coordinates are used. The factor $8\pi^2$ is obtained by explicitly integrating out

the three external orientational degrees of the solute. Therefore, z_A is not only independent of the position of the solute, but also on its orientation.

The intramolecular configurational partition function of A in solution can be concisely written as

$$z_A^l = \int d\mathbf{q}_A \exp(-\beta W_{A|l}(\mathbf{q}_A) \tag{4}$$

where we have made use of the solvent-averaged potential of mean force (PMF) $W_{A|l}(\mathbf{q}_A)$ acting on a particular conformation of the solute in the usual manner,[27]

$$\exp(-\beta W_{A|l}(\mathbf{q}_A)) = 1/Z_N \int d\mathbf{r}_N \exp(-\beta U(\mathbf{q}_A, \mathbf{r}_N)) \tag{5}$$

Here, Z_N is the configurational partition function of the pure liquid (e.g., water), which is an additive offset to the PMF. The $\mathbf{r}_N$ denote the cartesian coordinates of all solvent molecules, the $\mathbf{q}_A$ the $3N_A - 6$ internal coordinates of the solute. Any Jacobian factors are included in $\mathbf{q}_A$. Definitions Eqs. 4 and 5 are given for completeness and are not required further.

Combining Eqs. 2 and 3 and rearranging the terms slightly leads to

$$\mu_A^l = -k_B T \ln \frac{8\pi^2}{C^l \prod_i \lambda_i^3} - k_B T \ln z_A^l \tag{6}$$

The first term on the right-hand-side of Eq. 6 contains the positional (number density C^l) and orientational ($8\pi^2$) contributions to the chemical potential, as well as the momentum contribution via the de Broglie wavelengths λ_i. It is *completely independent* of the nature of the solute *or* solvent, which enters through the second term $-k_B T \ln z_A^l$. This second term corresponds to Ben-Naim's *pseudochemical* potential.[26]

Eq. 2 or 6 becomes a standard chemical potential μ^0 upon changing the actual number density C^l to the number density of the respective standard state, e.g., $C^0 = 1/1661$ particles/Å^3 for a 1 molar standard concentration. Note that the introduction of a standard state also is completely agnostic to the nature of the system. Next, we write out

explicitly the standard chemical potentials of A in the gas phase and in solution

$$\mu_A^{0,g} = -k_B T \ln \frac{8\pi^2}{C^{0,g} \prod_i \lambda_i^3} - k_B T \ln z_A^g \tag{7}$$

$$\mu_A^{0,l} = -k_B T \ln \frac{8\pi^2}{C^{0,l} \prod_i \lambda_i^3} - k_B T \ln z_A^l \tag{8}$$

where we deliberately leave the choice of standard concentration open (more below). The intramolecular configurational gas phase partition function of A is given by $z_A^l = \int d\mathbf{q}_A \exp(-\beta U(\mathbf{q}_A))$, with $U(\mathbf{q_A})$ the intramolecular potential energy of just the solute.

Thus, we can finally express ΔA^0_{solv} as

$$\Delta A^0_{\text{solv}} = \mu_A^{0,l} - \mu_A^{0,g} = -k_B T \ln \frac{C^{0,g}}{C^{0,l}} - k_B T \ln \frac{z_A^l}{z_A^g} \tag{9}$$

The first term on the right-hand-side of Eq. 9 just depends on the choice of standard states; in the Ben-Naim convention, $C^{0,g} = C^{0,l} = 1$ mol/L and, hence, it vanishes. The second term $-k_B T \ln z_A^l / z_A^g$ is what we calculate in an alchemical free energy simulation when decoupling the solute from the solvent. Following Ben-Naim, it can be interpreted as the free energy of transferring the solute from a fixed position in the gas phase to a fixed position in solution. Whether this is indeed a fixed position with only the solvent molecules moving around it, or the solute being able to move freely in the solvent and gas phase environment is irrelevant, since in both states the environment is isotropic. The key observation is that the term has no volume dependency to begin with. Any volumes or number densities entering the final expression are those of the respective standard states. Therefore, the application of our anchor restraint on a particular solute atom has no influence on the calculated ΔA^0_{solv}.

In passing, we point out that in our derivation we have also factored out the three external orientational degrees of the solute (the factor $8\pi^2$ which cancels from Eq. 9) from q'_A and z_A. This shows that ΔA^0_{solv} also does not depend on the solute's orientation. If we, as planned

for future work, will restrain these orientational degrees of the solute, then integrating over them will not give $8\pi^2$ but some other value $\Theta \leq 8\pi^2$. It is trivial to ensure that the gas phase and solvent Θ^g and Θ^l contributions are identical and, thus, will cancel from $\Delta A^0_{\mathrm{solv}}$. In other words, the solvation free energy of a solute A is also independent of orientational restraints.

Additional numerical consistency checks

Endpoint validation

At the end states $\lambda = 0$ and $\lambda = 1$, a correctly implemented alchemical coupling / decoupling scheme should result in one solute being fully decoupled from the environment, thereby behaving as an ideal gas-phase species. To verify that our implementation reproduces this behavior, we performed a consistency check based on energy decomposition. Specifically, given a configuration from an equilibrated trajectory, we evaluate the potential energy in three ways: (i) using the full 4D-coupling/decoupling framework, denoted as E_{4D}; (ii) by evaluating the energy of solute 1 together with the solvent, with solute 2 removed from all interactions (denoted $E^{\lambda=0}_{\mathrm{S1+solv}}$); and (iii) by evaluating solute 2 in isolation in the gas phase, denoted $E^{\lambda=0}_{\mathrm{S2}}$. If the coupling/decoupling scheme is implemented correctly, these contributions should be additive at $\lambda = 0$, such that:

$$E^{\lambda=0}_{4D} = E^{\lambda=0}_{\mathrm{S1+solv}} + E^{\lambda=0}_{S2}.$$

An analogous validation can be performed at $\lambda = 1$ by exchanging the roles of S1 and S2, i.e., with solute 2 coupled to the solvent and solute 1 treated as a gas-phase species. For illustration, we report numerical results for all starting coordinates for all investigated solute pairs in Table S2.

Table S2: Endpoint validation at $\lambda = 0$ and $\lambda = 1$. All values are given in kcal/mol.

S1	S2	$E^{\lambda=0}_{\text{S1+solv}}$	$E^{\lambda=0}_{S2(gas)}$	$E^{\lambda=0}_{\text{S1+solv}} + E^{\lambda=0}_{S2(gas)}$	$E^{\lambda=0}_{4D}$	error
Ethane	Water	-448,616.93	-966.53	-449,583.46	-449,583.46	$\leq 10^{-9}$
Ethane	Methanol	-448,616.93	-2,118.89	-450,735.82	-450,735.82	$\leq 10^{-12}$
Toluene	Phenol	-450,487.63	-6,122.38	-456,610.00	-456,610.00	$\leq 10^{-10}$
Ethanol	Methane	-450,060.12	-1,742.57	-451,802.69	-451,802.69	$\leq 10^{-9}$
Phenol	Ethane	-450,927.61	-2,964.89	-453,892.50	-453,892.50	$\leq 10^{-9}$
Methane	Ethane	-450,307.42	-2,964.83	-453,272.25	-453,272.25	$\leq 10^{-9}$
Methanol	Ethanol	-449,621.18	-3,342.68	-452,963.86	-452,963.86	$\leq 10^{-9}$
Methylthiol	Ethane	-449,659.64	-2,964.87	-452,624.51	-452,624.51	$\leq 10^{-10}$
Acetone	Acetamide	-448,816.73	-3,603.41	-452,420.14	-452,420.14	$\leq 10^{-9}$
Toluene	Tetrahydrofuran	-450,487.63	-5,234.41	-455,722.04	-455,722.04	$\leq 10^{-9}$
S1	S2	$E^{\lambda=1}_{S1(gas)}$	$E^{\lambda=1}_{\text{S2+solv}}$	$E^{\lambda=1}_{\text{S1(gas)}} + E^{\lambda=1}_{S2+solv}$	$E^{\lambda=1}_{4D}$	error
Ethane	Water	-2,964.91	-446,600.41	-449,565.32	-449,565.32	$\leq 10^{-9}$
Ethane	Methanol	-2,964.91	-447,758.83	-450,723.74	-450,723.74	$\leq 10^{-9}$
Toluene	Phenol	-6,918.59	-449,388.92	-456,307.50	-456,307.50	$\leq 10^{-9}$
Ethanol	Methane	-3,342.57	-448,349.64	-451,692.21	-451,692.21	$\leq 10^{-9}$
Phenol	Ethane	-6,122.42	-447,642.37	-453,764.79	-453,764.79	$\leq 10^{-9}$
Methane	Ethane	-1,742.66	-451,317.53	-453,060.19	-453,060.19	$\leq 10^{-9}$
Methanol	Ethanol	-2,118.79	-450,595.87	-452,714.66	-452,714.66	$\leq 10^{-9}$
Methylthiol	Ethane	-1,976.82	-450,633.58	-452,610.40	-452,610.40	$\leq 10^{-9}$
Acetone	Acetamide	-4,061.91	-448,275.03	-452,336.95	-452,336.95	$\leq 10^{-12}$
Toluene	Tetrahydrofuran	-6,918.59	-448,383.08	-455,301.67	-455,301.67	$\leq 10^{-9}$

Three-state cycle closure

As a complementary test, we combine our RSFE and ASFE results to demonstrate cycle closer for a three-state thermodynamic cycle. We construct a three state cycle between methanol, ethane and water as illustrated in Fig. S1:

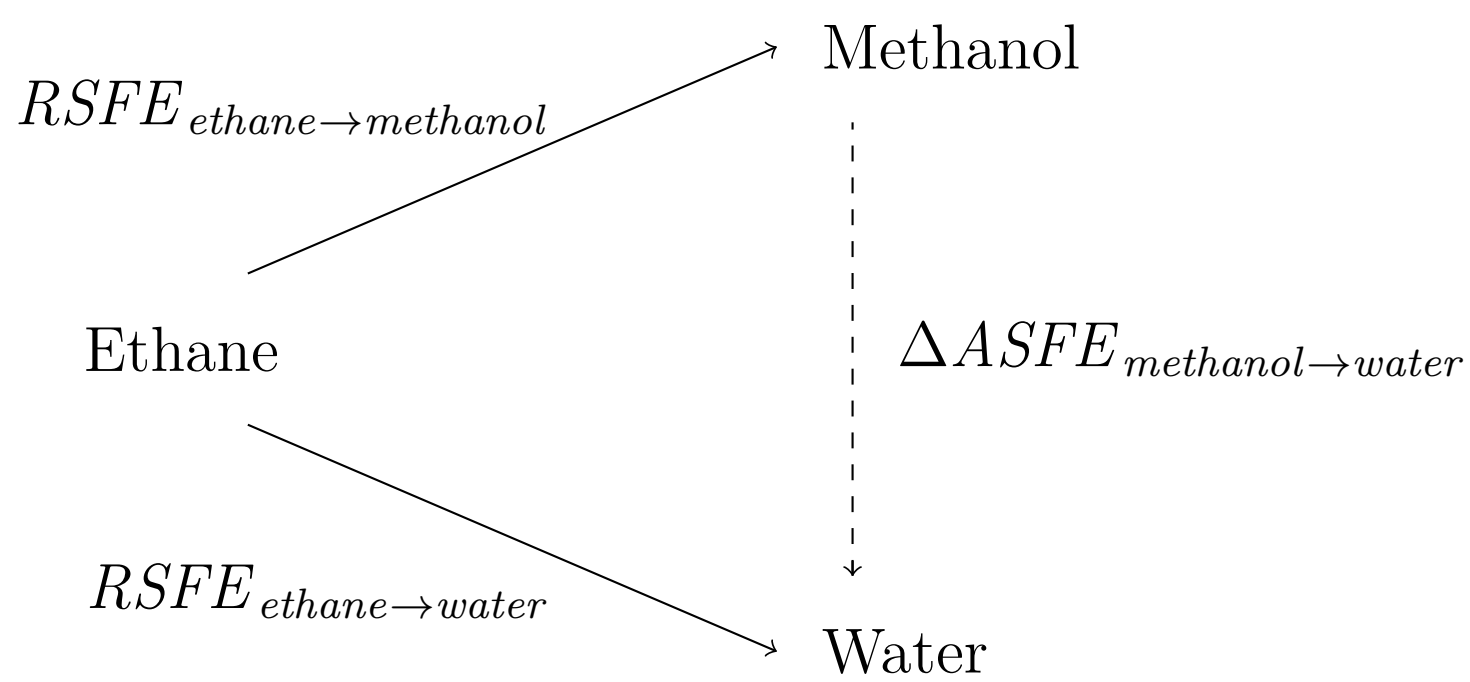

Figure S1: Three-state cycle from RSFE and ASFE results.

We compute the relative solvation free energy (RSFE) for the transformation methanol → water along two independent thermodynamic paths. In the first approach, the RSFE is obtained indirectly as a difference of relative solvation free energies referenced to a common intermediate, ethane, i.e., $RSFE_{methanol\rightarrow water} = RSFE_{ethane\rightarrow water} - RSFE_{ethane\rightarrow methanol}$. In the second approach, the RSFE is computed directly from absolute solvation free energies (ASFE) as $RSFE_{methanol\rightarrow water} = ASFE_{water} - ASFE_{methanol}$. The following cycle-closure condition has to hold between these two formulations:

$$ASFE_{water} - ASFE_{methanol} = RSFE_{ethane\rightarrow water} - RSFE_{ethane\rightarrow methanol},$$

In Table S3, we report the ASFE and RSFE results from the main manuscript and assess cycle closure by evaluating the mean and standard deviation of the differences $\Delta \text{RSFE}_{methanol\rightarrow water}$ and $\Delta \text{ASFE}_{methanol\rightarrow water}$. The observed cycle closure error of 0.22 kcal/mol with a standard deviation of ±0.35 kcal/mol is slightly larger than that obtained from the direct RSFE

calculations, but still within the statistical error estimate.

Table S3: $\Delta ASFE_{methanol \rightarrow water}$ refers to $ASFE_{water} - ASFE_{methanol}$ and $\Delta RSFE_{methanol \rightarrow water}$ refers to $RSFE_{ethane \rightarrow water} - RSFE_{ethane \rightarrow methanol}$. Cycle closure corresponds to $\Delta ASFE_{methanol \rightarrow water} - \Delta RSFE_{methanol \rightarrow water}$. All values are given in kcal/mol. Standard deviations were computed using Gaussian error propagation.

	$(\Delta)\Delta G$
$RSFE_{ethane \rightarrow water}$	-8.85 ± 0.19
$RSFE_{ethane \rightarrow methanol}$	-6.94 ± 0.26
$\Delta RSFE_{methanol \rightarrow water}$	-1.91 ± 0.33
$ASFE_{water}$	-6.48 ± 0.05
$ASFE_{methanol}$	-4.79 ± 0.10
$\Delta ASFE_{methanol \rightarrow water}$	-1.69 ± 0.11
Cycle closure	0.22 ± 0.35

Pairwise overlap matrix

In Figs. S2 and S3, we present pairwise BAR overlap matrices for the alchemical transformations ethane → methanol and acetone → acetamide, respectively. Since the pairwise BAR formulation was employed, non-zero overlap values are present only between neighboring λ-windows. For the transformation from an apolar solute (ethane) to a polar solute (methanol), the overlap between adjacent λ-states shows a modest decrease with increasing λ, consistent with the progressive decoupling of ethane and concomitant coupling of methanol to the solvent environment. Overall, the equidistant λ-schedule appears to resolve the free energy landscape smoothly, without evidence of abrupt discontinuities. A similar behavior is observed for the acetone → acetamide transformation, where the overlap between neighboring λ-states remains within a relatively uniform range across the alchemical pathway. We emphasize that the λ-schedules used in this work were not subject to systematic optimization. Accordingly, we do not claim that the chosen discretization is optimal for all solute pairs; such optimization would likely require system-specific tuning of the λ distribution.

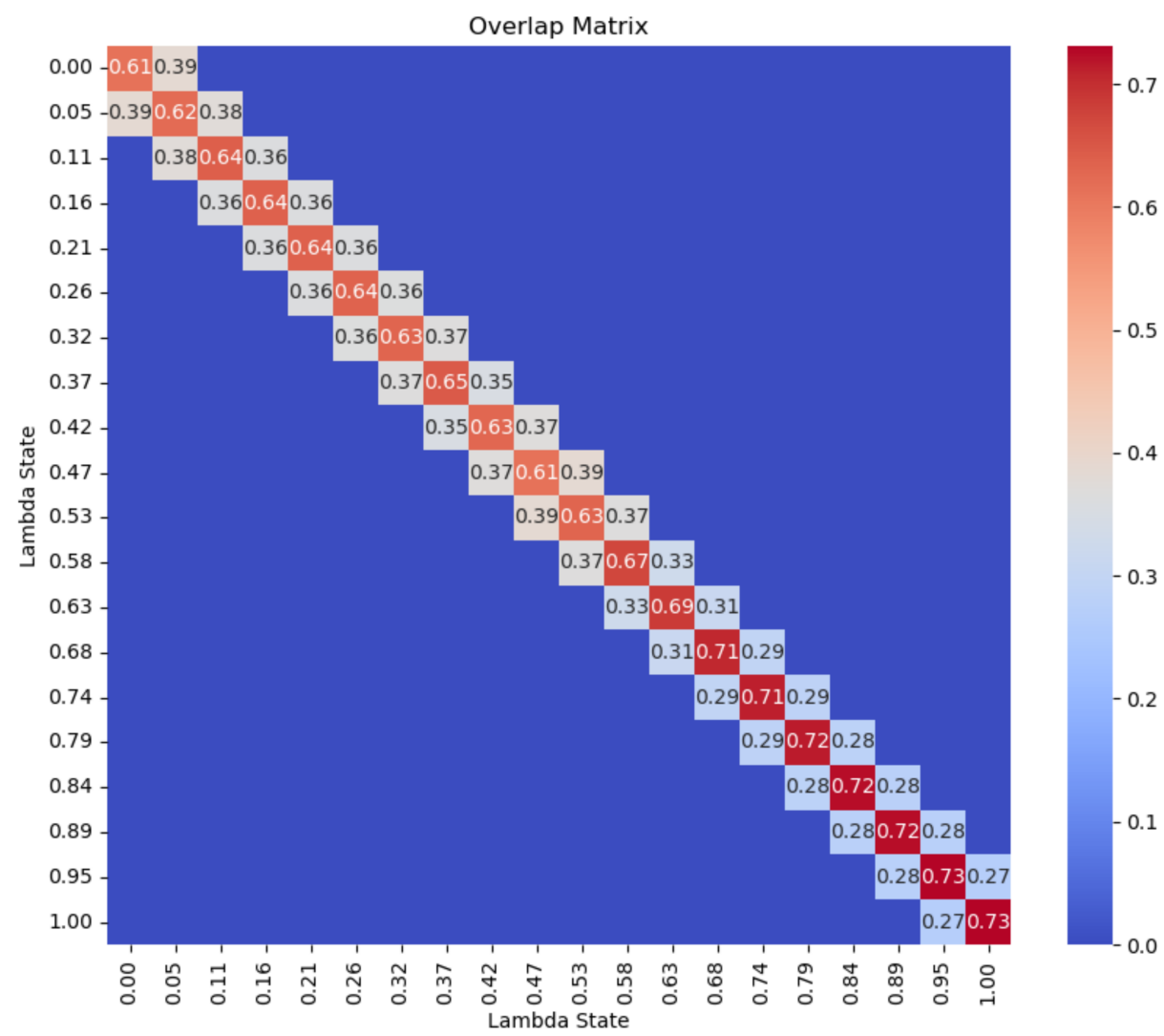

Figure S2: Pairwise overlap matrix for one exemplary ethane / methanol RSFE run.

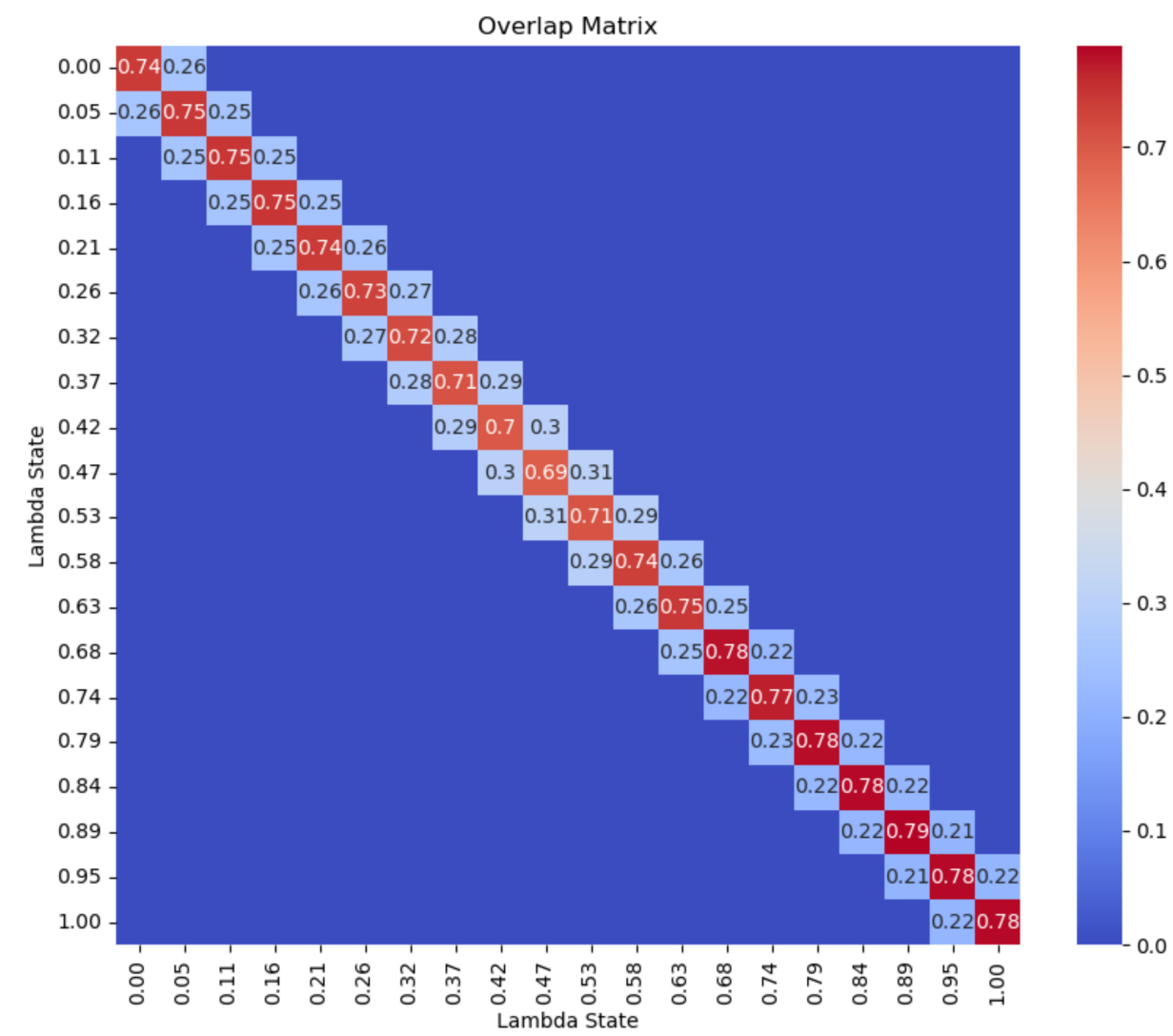

Figure S3: Pairwise overlap matrix for one exemplary acetone / acetamide RSFE run.

Additional Tables

Complete numerical results

We list all numerical ASFE results for all three repeats and all solutes in Table S4, as well as numerical RSFE results for all three repeats and all solute pairs in Table S5. To test for statistical noise and account for statistical outliers, we ran two additional runs for the solute pair acetone → acetamide. To be consistent with the general protocol for all solute pairs, all mean values and standard deviations in Table S5 and the main manuscript were computed from the first three runs only. The results for Run4 and Run5 for acetone → acetamide including the mean value and standard deviation considering all 5 runs are given as a subscript in Table S5.

Simulation Systems

Table S6 reports the number of water molecules as well as the instantaneous box size after 100 ps equilibration with the MACE-OFF23(S) model for each solute S for which we performed ASFE calculations. The same boxes were also used to generate initial coordinates

Table S4: All ASFE results for all three repeats per solute.

	Run1	Run2	Run3	Mean	Std
Water	-6.54	-6.47	-6.44	-6.48	0.05
Methane	2.03	2.01	2.07	2.03	0.03
Ethane	2.23	2.39	2.14	2.26	0.13
Methanol	-4.68	-4.80	-4.88	-4.79	0.10
Ethanol	-4.14	-4.25	-4.37	-4.25	0.12
Toluene	-1.26	-1.19	-1.34	-1.26	0.07
Phenol	-6.06	-5.81	-6.00	-5.96	0.13
Acetone	-2.98	-3.22	-2.90	-3.03	0.17
Acetamide	-9.37	-9.34	-9.62	-9.44	0.15
Tetrahydrofuran	-3.37	-3.03	-3.32	-3.24	0.18
Methylthiol	0.27	0.40	0.18	0.28	0.11

Table S5: All RSFE results for all three repeats per solute pair.

S1	S2	Run1	Run2	Run3	Mean	Std
Ethane	Water	-8.83	-9.05	-8.67	-8.85	0.19
Water	Ethane	8.62	8.71	8.94	8.76	0.17
Ethane	Methanol	-6.64	-7.10	-7.09	-6.94	0.26
Toluene	Phenol	-4.83	-4.65	-4.55	-4.68	0.14
Ethanol	Methane	6.14	6.31	6.61	6.35	0.24
Phenol	Ethane	8.14	8.20	8.19	8.18	0.03
Methane	Ethane	0.07	0.30	0.22	0.20	0.12
Methanol	Ethanol	0.85	0.35	0.29	0.50	0.31
Methylthiol	Ethane	2.10	1.95	2.02	2.02	0.07
Acetone	Acetamide	-6.56	-6.52	-6.04	-6.37*	0.29*
Toluene	Tetrahydrofuran	-2.09	-1.95	-2.19	-2.07	0.12

* Acetone → Acetamide Run4: -6.69, Run5: -6.40, Mean(Run1 - Run5): -6.44, Std(Run1 - Run5) = 0.25

for the RSFE calculations. Thus, for any solute pair $S1 \rightarrow S2$, the information given for S1 in the table indicates the initial box size from which the RSFE calculation were started.

Table S6: Details of the simulation systems used in the ASFE and RSFE calculations.

Solute	# water molecules	Box size
Water	437	22.7 Å
Methane	444	23.0 Å
Ethane	441	22.8 Å
Methanol	443	22.8 Å
Ethanol	442	22.9 Å
Toluene	439	23.0 Å
Phenol	440	22.9 Å
Methylthiol	443	22.8 Å
Acetone	440	22.8 Å
Acetamide	439	22.7 Å
Tetrahydrofuran	440	22.9 Å

Comparison to force field studies

Table S7 reports all ASFEs computed with (1) the 4D-decoupling approach using the MACE-OFF23(S) model (this work and Ref. 19), (2) CGenFF, as reported in Ref. 28, and (3) ABCG2, as reported in Ref. 29. These are the data used to calculate the MAE and RMSE values reported in the main manuscript.

Table S7: ASFE results ΔG_{solv} in kcal/mol obtained with 4D-decoupling and MACE-OFF23(S) in this work, as well as the corresponding values from two force field studies. The experimental values are taken from the FreeSolv[30] or the Minnesota solvation database.[31] The 4D-decoupling results for compounds marked with a star * are from Ref. 19. NA indicates that the primary source does not report this value.

	4D-decoupling MACE-OFF23(S)	CGenFF[28]	ABCG2[29]	Experiment
Water*	-6.48 ± 0.05	N/A	N/A	-6.30
Methane*	+2.03 ± 0.03	+2.42 ± 0.05	+2.27 ± 0.03	2.00
Ethane*	+2.26 ± 0.13	+2.27 ± 0.04	+2.24 ± 0.02	1.83
Methanol*	-4.79 ± 0.10	-4.63 ± 0.06	-4.65 ± 0.05	-5.10
Ethanol*	-4.25 ± 0.12	-4.49 ± 0.06	-4.68 ± 0.05	-5.00
Toluene*	-1.26 ± 0.07	+0.04 ± 0.06	-0.45 ± 0.09	-0.90
Phenol*	-5.96 ± 0.13	-4.66 ± 0.04	-6.09 ± 0.08	-6.60
Acetone	-3.03 ± 0.17	-4.37 ± 0.04	-3.30 ± 0.03	-3.80
Acetamide	-9.44 ± 0.15	-7.64 ± 0.07	-9.32 ± 0.09	-9.71
Tetrahydrofuran	-3.24 ± 0.18	-2.46 ± 0.05	-2.67 ± 0.08	-3.47
Methylthiol	+0.28 ± 0.11	-0.15 ± 0.04	-1.17 ± 0.09	-1.20
MAE	0.45	0.83	0.41	
RMSD	0.66	1.11	0.45	

References

(1) Plé, T.; Lagardère, L.; Piquemal, J.-P. Force-field-enhanced neural network interactions: from local equivariant embedding to atom-in-molecule properties and long-range effects. *Chemical Science* **2023**, *14*, 12554–12569.

(2) Batatia, I.; Kovács, D. P.; Simm, G. N. C.; Ortner, C.; Csányi, G. MACE: Higher Order Equivariant Message Passing Neural Networks for Fast and Accurate Force Fields. 2022.

(3) Kovács, D. P.; Moore, J. H.; Browning, N. J.; Batatia, I.; Horton, J. T.; Pu, Y.; Kapil, V.; Witt, W. C.; Magdău, I.-B.; Cole, D. J.; Csányi, G. MACE-OFF: Short-Range Transferable Machine Learning Force Fields for Organic Molecules. *Journal of the American Chemical Society* **2025**, *147*, 17598–17611, PMID: 40387214.

(4) Schütt, K. T.; Kindermans, P.-J.; Sauceda, H. E.; Chmiela, S.; Tkatchenko, A.; Müller, K.-R. SchNet: A continuous-filter convolutional neural network for modeling quantum interactions. *arXiv* **2017**,

(5) Eichenberger, A. P.; Allison, J. R.; Dolenc, J.; Geerke, D. P.; Horta, B. A. C.; Meier, K.; Oostenbrink, C.; Schmid, N.; Steiner, D.; Wang, D.; van Gunsteren, W. F. GROMOS++ Software for the Analysis of Biomolecular Simulation Trajectories. *Journal of Chemical Theory and Computation* **2011**, *7*, 3379–3390.

(6) Schmid, N.; Christ, C. D.; Christen, M.; Eichenberger, A. P.; van Gunsteren, W. F. Architecture, implementation and parallelisation of the GROMOS software for biomolecular simulation. *Computer Physics Communications* **2012**, *183*, 890–903.

(7) Devereux, C.; Smith, J. S.; Huddleston, K. K.; Barros, K.; Zubatyuk, R.; Isayev, O.; Roitberg, A. E. Extending the Applicability of the ANI Deep Learning Molecular Potential to Sulfur and Halogens. *J. Chem. Theory Comput.* **2020**, *16*, 4192–4202.

(8) Vanommeslaeghe, K.; Hatcher, E.; Acharya, C.; Kundu, S.; Zhong, S.; Shim, J.; Darian, E.; Guvench, O.; Lopes, P.; Vorobyov, I.; Mackerell Jr., A. D. CHARMM general force field: A force field for drug-like molecules compatible with the CHARMM all-atom additive biological force fields. *Journal of Computational Chemistry* **2010**, *31*, 671–690.

(9) Vanommeslaeghe, K.; MacKerell, A. D. J. Automation of the CHARMM General Force Field (CGenFF) I: Bond Perception and Atom Typing. *Journal of Chemical Information and Modeling* **2012**, *52*, 3144–3154, PMID: 23146088.

(10) Vanommeslaeghe, K.; Raman, E. P.; MacKerell, A. D. J. Automation of the CHARMM General Force Field (CGenFF) II: Assignment of Bonded Parameters and Partial Atomic Charges. *Journal of Chemical Information and Modeling* **2012**, *52*, 3155–3168, PMID: 23145473.

(11) Weber, J. L.; Guha, R. D.; Agarwal, G.; Wei, Y.; Fike, A. A.; Xie, X.; Stevenson, J.; Santra, B.; Friesner, R. A.; Leswing, K.; Halls, M. D.; Abel, R.; Jacobson, L. D. Efficient Long-Range Machine Learning Force Fields for Liquid and Materials Properties. 2025.

(12) Wang, J.; Wolf, R. M.; Caldwell, J. W.; Kollman, P. A.; Case, D. A. Development and testing of a general amber force field. *Journal of Computational Chemistry* **2004**, *25*, 1157–1174.

(13) Wang, J.; Wang, W.; Kollman, P. A.; Case, D. A. Automatic atom type and bond type perception in molecular mechanical calculations. *Journal of Molecular Graphics and Modelling* **2006**, *25*, 247–260.

(14) Zou, J.; Tian, C.; Simmerling, C. Blinded prediction of protein–ligand binding affinity using Amber thermodynamic integration for the 2018 D3R grand challenge 4. *Journal of Computer-Aided Molecular Design* **2019**, *33*, 1021–1029.

(15) Maier, J. A.; Martinez, C.; Kasavajhala, K.; Wickstrom, L.; Hauser, K. E.; Simmer-

ling, C. ff14SB: Improving the Accuracy of Protein Side Chain and Backbone Parameters from ff99SB. *Journal of Chemical Theory and Computation* **2015**, *11*, 3696–3713.

(16) Plé, T.; Adjoua, O.; Benali, A.; Posenitskiy, E.; Villot, C.; Lagardère, L.; Piquemal, J.-P. A Foundation Model for Accurate Atomistic Simulations in Drug Design. *ChemRxiv* **2025**,

(17) Harry Moore, J.; Cole, D. J.; Csányi, G. Computing Solvation Free Energies of Small Molecules with Experimental Accuracy. *Journal of the American Chemical Society* **2026**, *148*, 4928–4937.

(18) Tkaczyk, S.; Langer, T.; Wieder, M.; Rizzi, A.; Boresch, S. Repeatability of Relative Free Energy Calculations in Solution with ANI-2x and MACE-OFF23. *Journal of Chemical Theory and Computation* **2025**, *22*, 231–239.

(19) Picha, A. K.; Tkaczyk, S.; Langer, T.; Wieder, M.; Boresch, S. Architecture-Independent Absolute Solvation Free Energy Calculations with Neural Network Potentials. *The Journal of Physical Chemistry Letters* **2025**, *16*, 12080–12086, PMID: 41217401.

(20) Crha, R.; Poliak, P.; Gillhofer, M.; Oostenbrink, C. Alchemical Free-Energy Calculations at Quantum-Chemical Precision. *The Journal of Physical Chemistry Letters* **2025**, *16*, 863–869.

(21) Karwounopoulos, J.; Wu, Z.; Tkaczyk, S.; Wang, S.; Baskerville, A.; Ranasinghe, K.; Langer, T.; Wood, G. P. F.; Wieder, M.; Boresch, S. Insights and Challenges in Correcting Force Field Based Solvation Free Energies Using a Neural Network Potential. *The Journal of Physical Chemistry B* **2024**, *128*, 6693–6703.

(22) Karwounopoulos, J.; Bieniek, M.; Wu, Z.; Baskerville, A. L.; König, G.; Cossins, B. P.; Wood, G. P. F. Evaluation of Machine Learning/Molecular Mechanics End-State Cor-

rections with Mechanical Embedding to Calculate Relative Protein–Ligand Binding Free Energies. *Journal of Chemical Theory and Computation* **2025**, *21*, 967–977.

(23) Xie, X.; Weber, J. L.; Svensson, M.; Johnston, R. C.; Harder, E. D.; Jacobson, L. D. Accurate Hydration Free Energy Calculations for Diverse Organic Molecules With a Machine Learning Force Field. *Journal of Chemical Theory and Computation* **2026**,

(24) Sabanés Zariquiey, F.; Galvelis, R.; Gallicchio, E.; Chodera, J. D.; Markland, T. E.; De Fabritiis, G. Enhancing Protein–Ligand Binding Affinity Predictions Using Neural Network Potentials. *Journal of Chemical Information and Modeling* **2024**, *64*, 1481–1485.

(25) Sabanés Zariquiey, F.; Farr, S. E.; Doerr, S.; De Fabritiis, G. QuantumBind-RBFE: Accurate Relative Binding Free Energy Calculations Using Neural Network Potentials. *Journal of Chemical Information and Modeling* **2025**, *65*, 4081–4089.

(26) Ben-Naim, A.; Marcus, Y. Solvation thermodynamics of nonionic solutes. *The Journal of Chemical Physics* **1984**, *81*, 2016–2027.

(27) Roux, B.; Simonson, T. Implicit solvent models. *Biophysical Chemistry* **1999**, *78*, 1–20.

(28) Karwounopoulos, J.; Kaupang, Å.; Wieder, M.; Boresch, S. Calculations of Absolute Solvation Free Energies with Transformato—Application to the FreeSolv Database Using the CGenFF Force Field. *Journal of Chemical Theory and Computation* **2023**, *19*, 5988–5998.

(29) He, X.; Man, V. H.; Yang, W.; Lee, T.-S.; Wang, J. ABCG2: A Milestone Charge Model for Accurate Solvation Free Energy Calculation. *Journal of Chemical Theory and Computation* **2025**, *21*, 3032–3043.

(30) Mobley, D. L.; Guthrie, J. P. FreeSolv: a database of experimental and calculated

hydration free energies, with input files. *Journal of Computer-Aided Molecular Design* **2014**, *28*, 711–720.

(31) Marenich, A. V.; Kelly, C. P.; Thompson, J. D.; Hawkins, G. D.; Chambers, C. C.; Giesen, D. J.; Winget, P.; Cramer, C. J.; Truhlar, D. G. Minnesota Solvation Database (MNSOL) version 2012. *Data Repository for the University of Minnesota (DRUM)* **2020**,
